# The oral microbiome in relation to systemic maternal conditions and pregnancy complications: a systematic review and meta-analysis

**DOI:** 10.1080/20002297.2026.2717774

**Published:** 2026-08-26

**Authors:** Jiawei Xu, Cong Li, Weifen Wu, Rongrong Zheng, Yongfeng Wu, Huan Zhou

**Affiliations:** a Stomatology Hospital, School of Stomatology, Zhejiang University School of Medicine, Zhejiang Provincial Clinical Research Center for Oral Diseases, Zhejiang Key Laboratory of Oral Biomedical, Engineering Research Center of Oral Biomaterials and Devices of Zhejiang Province, Hangzhou, China; b Department of Obstetrics and Gynecology, Affiliated Hangzhou First People's Hospital, School of Medicine, Westlake University, Hangzhou, China

**Keywords:** Oral microbiome, pregnancy complications, maternal systemic conditions, microbial diversity, high-throughput sequencing, meta-analysis

## Abstract

**Background:**

Alterations in the oral microbiome have been associated with systemic diseases, but evidence regarding maternal conditions and pregnancy complications remains limited. This systematic review evaluated these associations.

**Methods:**

PubMed, Web of Science, and Embase were searched for studies using high-throughput sequencing to profile the oral microbiome in pregnant women with systemic diseases or adverse pregnancy outcomes. Random-effects meta-analyses were performed for alpha diversity indices, while methodological heterogeneity precluded quantitative synthesis of beta diversity and taxonomic abundance.

**Results:**

Twenty-four studies were included, with 11 contributing to the meta-analysis. Gestational diabetes mellitus (GDM) was associated with lower Observed richness (SMD = −0.29, 95% CI: −0.55 to −0.03), whereas low birth weight (LBW) was associated with higher Chao1 (SMD = 0.46, 95% CI: 0.03–0.88). No significant alpha diversity differences were observed for pre-eclampsia or preterm birth. Differences in beta diversity and taxonomic abundance were reported across several conditions, although findings were heterogeneous.

**Conclusions:**

Oral microbial richness may be associated with GDM and LBW, but associations with other pregnancy complications remain uncertain. Standardized, large-scale prospective studies are needed.

## Introduction

Pregnancy is a unique physiological state requiring extensive adaptations across multiple organ systems. Concurrently, the maternal microbiota undergoes substantial alterations across multiple body sites in response to these physiological adaptations [1,2]. Although the maternal microbiota undergoes dynamic changes during pregnancy, excessive alterations in microbial diversity, community composition and the overgrowth of potentially pathogenic microorganisms may disrupt microbial homeostasis, resulting in dysbiosis and an increased risk of adverse health outcomes [3,4]. Furthermore, the maternal microbiota is an important source of neonatal microbial colonization and plays a critical role in early-life development and immune system maturation [5,6].

Recent advances in high-throughput sequencing technologies have fundamentally reshaped our understanding of microbial ecosystems, revealing their remarkable complexity and dynamism [7], while enabling quantification of microbial abundance, identification of unculturable taxa and characterization of complex clinical specimens. Notably, these technologies have demonstrated that the placental microbiome shares greater taxonomic similarity with the oral microbiome than with the vaginal microbiome [8]. Growing evidence suggests that oral dysbiosis is associated with obstetric complications, potentially through complex inflammatory, immunological and metabolic mechanisms [9]. Such dysbiotic states are commonly characterized using alpha and beta diversity metrics, where alpha diversity captures within-sample species richness and evenness, while beta diversity reflects differences in microbial community composition between samples.

The human oral cavity harbors a complex microbial ecosystem comprising more than 700 identified bacterial species [10,11]. Maintaining oral microecological homeostasis is essential not only for oral health but also for systemic health. Evidence regarding the association between the oral microbiota and pregnancy-related conditions remains limited and fragmented. To date, most systematic reviews have focused on specific pregnancy complications, such as pre-eclampsia, miscarriage and gestational diabetes mellitus (GDM), or on individual periodontal pathogens, including *Fusobacterium nucleatum* and *Porphyromonas gingivalis*. A comprehensive synthesis integrating multiple pregnancy-related conditions with both quantitative analyzes of microbial diversity and qualitative evaluation of microbial taxonomic composition remains lacking [12–16]. Meanwhile, existing narrative reviews examining microbiota changes during pregnancy have primarily focused on hormonal and immunological mechanisms, with less attention devoted to microbial diversity metrics [2,9]. Although Xiao et al. conducted a meta-analysis on pregnancy-related oral microbiota shifts and maternal conditions, variability in measurement metrics​ undermines the generalizability​ of their results [17]. In light of these limitations and the conflicting results reported to date, there is a pressing need for a comprehensive evaluation of the dynamic alterations in the oral microbiota during pregnancy and their associations with systemic diseases and adverse pregnancy outcomes.

This review aimed to systematically summarize the current evidence on oral microbiome alterations during pregnancy and their reported associations with adverse pregnancy and birth outcomes. Secondary outcomes assessed the consistency and reproducibility of findings across studies by examining reported changes in the relative abundance of microbial taxa (from phylum to species) associated with these conditions.

## Methods

This systematic review was conducted following standard systematic review methodology, and reported in accordance with the Preferred Reporting Items for Systematic Reviews and Meta-Analyzes (PRISMA) 2020 statement, and the protocol was registered in the PROSPERO (CRD420251076902) (https://www.crd.york.ac.uk/prospero/).

### Database sources and search methods

A systematic search of PubMed, Embase and Web of Science was conducted to identify peer-reviewed studies examining associations between the oral microbiota and maternal systemic conditions or pregnancy complications. We restricted our search to publications published between March 2015 and June 2025, as earlier studies employed outdated detection methodologies and heterogeneous protocols, which would have introduced substantial clinical heterogeneity and compromised the robustness of our pooled analysis. We utilized a combination of relevant keywords and controlled subject headings including ‘oral microbiota’, ‘bacterial diversity’, ‘systemic diseases’, ‘16S ribosomal RNA (rRNA) sequencing’, ‘pregnancy complications’ and ‘adverse pregnancy outcomes’. The detailed search strategies are provided in Appendix S1.

### Eligibility criteria

Eligible studies included case‒control, cross-sectional, retrospective and prospective cohort studies investigating oral microbiota characteristics in relation to maternal systemic conditions and pregnancy-related complications. Studies were selected according to the following predefined (population, intervention, comparison, outcome) PICO framework: (P) Population: Pregnant women, and where applicable, women of reproductive age included as healthy controls. (I) Intervention/exposure: Maternal systemic conditions (e.g. hypothyroidism, depression, type 2 diabetes mellitus (T2DM) and hypertension) or pregnancy-related complications or adverse birth outcomes (e.g. gestational diabetes mellitus (GDM), pre-eclampsia, preterm birth and low birth weight (LBW)). (C) Comparison: Women without the corresponding systemic condition, pregnancy complication, or adverse birth outcome. (O) Outcome: Oral microbiota characteristics, including alpha diversity, beta diversity, taxonomic composition and relative abundance. Additionally, only human studies published in English were included. We excluded literature reviews, editorials, letters, case series and studies that employed only culture-based methods or polymerase chain reaction (PCR) without high-throughput sequencing.

### Study selection

Two independent reviewers (Jiawei Xu and Cong Li) applied the above-stated eligibility criteria to screen titles and abstracts retrieved from electronic database searches. Potentially eligible articles then underwent full-text assessment. To minimize selection bias and ensure screening accuracy, any discrepancies between the two reviewers were resolved through discussion until consensus was reached. Furthermore, references and citations of the included articles were manually reviewed for additional articles.

### Data extraction

Data extraction was conducted in duplicate using a predefined form, with cross‑validation performed between authors (Jiawei Xu and Cong Li). The following data were extracted: study characteristics (author names, publication year, study design, sample type, sample collection time points, microbiome assessment method, sequencing technique, amplified region, sequencing platform and primary data source), group information (sample size per group, comparisons), microbial diversity (alpha and beta diversity), taxonomic profiles from phylum to species levels.

For studies that did not report alpha diversity indices (e.g. mean and standard deviation (SD)), quantitative data were extracted from graphical representations (box plots) using WebPlotDigitizer [18]. Medians and inter-quartile ranges were transformed to means and SDs using the Wan method (https://www.math.hkbu.edu.hk/~tongt/papers/median2mean.html).

### Quality assessment

Quality assessment of the included case‒control studies was performed using the Newcastle–Ottawa Scale (NOS) [19]. Methodological quality was performed across three core domains: selection (maximum 4 points), comparability (maximum 2 points) and outcome assessment (maximum 3 points). Based on the total NOS scores, all included studies were stratified into three quality tiers: high quality (7–9 points), moderate quality (4–6 points) and low quality (1–3 points) [20]. For longitudinal cohort and cross‑sectional studies, quality assessment was carried out using the NIH Quality Assessment Tool [21], which includes 14 items covering risk of bias, Type I and Type II errors, transparency and confounding factors. Items 6, 7 and 13 were excluded from the assessment of cross‑sectional studies due to inapplicability. For cohort studies, studies scoring ≥10 were classified as good quality, those scoring 6–9 as moderate and those scoring 0–5 as poor. Quality assessment was performed independently by two reviewers (Jiawei Xu and Cong Li), and any disagreements were resolved via consensus discussion.

### Microbiome-specific methodological risk of bias

To evaluate methodological reliability specific to microbiome research, four additional domains were assessed: microbial measurement reliability, sequencing and batch effect control, consistency of bioinformatic pipelines and appropriateness of statistical analysis. Each item was classified as having a low, unclear, or high risk of bias according to pre-specified criteria (Appendix S2). Quality assessment was performed independently by two reviewers (Jiawei Xu and Cong Li), with discrepancies resolved through discussion. The overall quality rating was downgraded for studies that were judged to have a high risk of bias in two or more microbiome-specific domains.

### Data synthesis

​A meta-analysis was performed for each alpha diversity index when at least two studies reported the same metric. Pooled standardized mean differences (SMDs) and 95% confidence intervals (CIs) were estimated using a random-effects model to account for between-study heterogeneity. Studies were weighted using the inverse-variance method. Consequently, the sample sizes of the case and control groups contributed to the estimation of within-study variances, which in turn determined the inverse-variance weights assigned to individual studies. When a study reported alpha diversity at multiple sampling time points, only one time point was included in the meta-analysis to avoid double-counting participants. Specifically, we selected the measurement obtained closest to outcome assessment, typically the final sampling point before delivery. For studies that collected multiple sample types from the same participants (e.g. saliva and supragingival plaque), data from the sample type with the largest sample size were included in the analysis. Between-study heterogeneity was quantified using the I^2^ statistic, with heterogeneity graded as low (25–49%), moderate (50–74%), or high (≥75%).

Publication bias was evaluated via funnel plots when more than 10 studies were included. Regarding beta diversity, we systematically summarized the principal outcomes from all included studies and provided a comparative analysis with detailed elaboration. Differences in bacterial relative abundance from phylum to species were summarized narratively. All statistical analyzes were conducted in Stata 17.0, with the metan command utilized for the meta-analysis. Results were considered statistically significant when the *P* value was less than 0.05.

## Results

### Study selection

**Figure 1.**
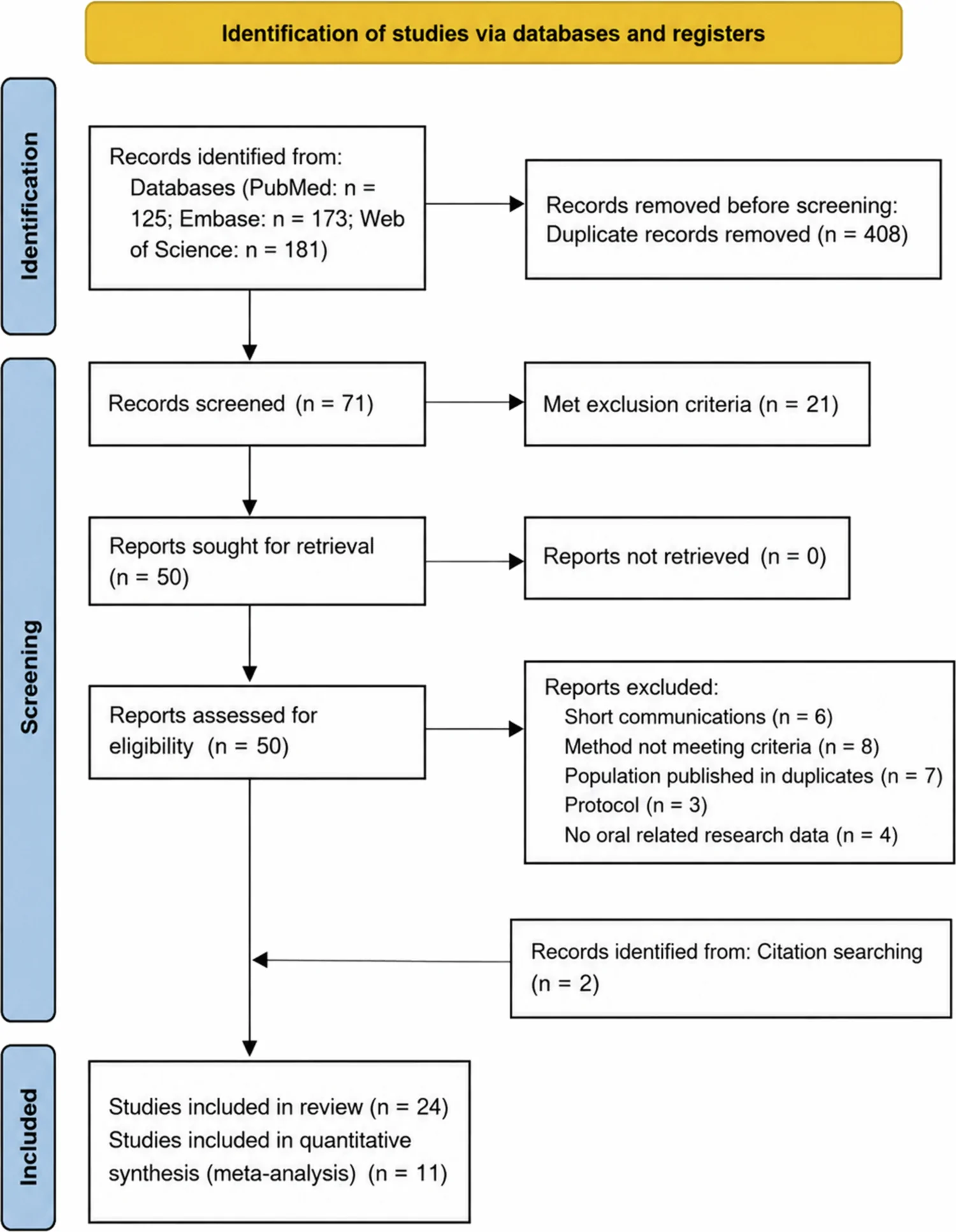
Flow diagram of study identification.

Our database search initially identified 479 records. After removal of 408 duplicates, 71 studies underwent a full eligibility assessment, of which 21 were excluded based on title and abstract screening. Finally, 24 studies were included [22–45], and data from 11 studies were extracted for meta-analytic pooling (Figure 1).

### Study characteristics

Characteristics of the included studies are summarized in Tables S1–S3. The 24 eligible studies were conducted across 11 countries, exhibiting significant geographic diversity: 12 (50.00%) focused on Western populations (e.g. USA, UK, Australia), 11 (45.83%) on East Asian populations (e.g. China, Korea) and 1 (4.17%) on South Africa. Publication dates ranged from 2018 to 2025. Sample sizes varied substantially, from small-scale cohorts (e.g. *n* = 11 for pre-eclampsia research) to large-scale investigations (e.g. *n* = 373 for depressive symptoms). Most studies set up case and healthy control groups for comparative analysis. Sampling strategies were heterogeneous, utilizing subgingival plaque, buccal swabs, sterile swabs, saliva and pooled oral mucosal samples. Specimens were collected across multiple gestational stages including the first, second and third trimesters, as well as the perinatal period (≤3 days postpartum) and up to 9 months postpartum; notably, the third trimester was the most frequently sampled time point (*n* = 18). Regarding study focus, five articles (20.83%) compared the oral microbiome between pregnant women with systemic diseases (hypothyroidism, mental health symptoms, T2DM and arterial hypertension) and healthy controls [22,23,25,33,40], while 19 articles (79.17%) explored associations between oral microbiota and adverse pregnancy outcomes, including GDM (*n* = 7), preterm birth (*n* = 4), pre-eclampsia (*n* = 3), LBW (*n* = 2) and other broad pregnancy complications (*n* = 3) [24,26–32,34–39,41–45].

Several analytical approaches were adopted to characterize microbial composition and diversity across included studies. Most studies (22/24) performed 16S rRNA gene sequencing on the Illumina platform, while the remaining two applied shotgun metagenomic sequencing. Among 16S-based investigations, the V3–V4 hypervariable region was the most frequently targeted (15/22), followed by the V4 region (22.7%), whereas 9.1% of studies sequenced other variable regions. Library preparation and sequencing were primarily conducted on four Illumina platforms: NovaSeq 6000, MiSeq, HiSeq 2500 and HiSeq 4000.

### Quality assessment

The quality assessment of the included case–control studies is shown in Table S4. For the 12 included case–control studies, the majority attained NOS scores of 6 or 7 points. All studies met the criteria for adequate case definition, appropriate control selection and definition and standardized exposure ascertainment with consistent methodologies for cases and controls. Cases representativeness, additional factors control and non-response rate were the most frequently omitted and thereby accounted for the lower quality score. Notably, the non-response rate domain was assigned a score of zero across all studies, as this methodological detail was not reported in the original publications.

The quality assessment of the cohort and cross-sectional studies is shown in Table S5. For cross-sectional studies included, the quality scores varied from 5 to 7, with all studies clearly stating research objectives, defining study populations and applying uniform inclusion/exclusion criteria. Key limitations included unreported sample size justification, non-evaluation of exposure gradients and inconsistent assessment of confounding variable adjustment. For cohort studies included, scores ranging from 6 to 8 and all studies maintained consistent definition and implementation of exposure and outcome measures. The main methodological shortcomings of cohort studies involved unreported formal sample size justification, unreported participation rates and non-blinded outcome assessment.

Overall, the included studies exhibited moderate to high methodological quality, with no studies excluded due to poor quality. The identified limitations were primarily related to incomplete confounding control and insufficient reporting of key study details, which should be considered when interpreting the pooled results.

### Microbiome-specific methodological risk of bias

Based on the risk of bias assessment for microbiome-specific methodologies (Table S6), the majority of included studies demonstrated low risk of bias​ across four key domains. For overall quality, the vast majority of studies (21/24) were rated ‘low’, encompassing all investigations of pre-eclampsia, hypothyroidism, mental health symptoms, T2DM and arterial hypertension. With regard to GDM, 5 studies (71.4%) were low overall risk, but 2 (28.6%) were unclear due to gaps in measurement reliability, batch control, or statistical reporting [42,45]. Besides, one study regarding adverse pregnancy outcomes was unclear overall risk of bias, due to incomplete methodological reporting, no batch effect control measures and lack of multiple comparison correction [31]. The comprehensive key justifications for risk of bias level of individual studies are shown in Appendix S3.

### Alpha diversity

Alpha diversity analysis enables the evaluation of compositional complexity within microbial communities, encompassing species richness and ecological heterogeneity. The present meta-analysis strictly included only primary studies with consistent outcome indicators and direct comparative designs. Among the alpha diversity metrics reported across eligible studies, the Shannon index was the most frequently adopted, followed by Chao1, Simpson index, ACE index and Observed richness.

#### Association between oral microorganisms during pregnancy and adverse pregnancy outcomes

Given the association between oral microbiota during pregnancy and adverse pregnancy outcomes, a total of 11 studies provided sufficient numerical or graphical data and were eligible for inclusion in the alpha diversity meta-analysis [24,26–28,34,35,37,39,42,44,45]. In comparisons between women with GDM and healthy controls, the GDM group exhibited significantly lower Observed richness (SMD = −0.29 [95%CI: −0.55, −0.03], *P* = 0.028, I^2^ = 23.9%). Notably, all other alpha diversity indices revealed no significant between-group differences (ACE index: SMD = −0.18 [95%CI: −1.70, 1.34], *P* = 0.814, I^2^ = 95%; Chao1 index: SMD = 0.04 [95%CI: −0.73, 0.80], *P* = 0.926, I^2^ = 87.1%; Simpson index: SMD = −0.22 [95%CI: −0.90, 0.46], *P* = 0.53, I^2^ = 84.2%; Shannon index: SMD = −0.08 [95%CI: −0.26, 0.1], *P* = 0.386, I^2^ = 0.0%). Detailed results are presented in Figure S1 and Table 1. Three studies examined alpha diversity in relation to LBW. The pooled analysis showed a significantly higher Chao1 index​ in the LBW group compared to controls (SMD = 0.46 [95%CI: 0.03, 0.88], *P* = 0.035), with no heterogeneity (I^2^ = 0.0%, *P* = 0.438). However, no significant difference was observed for the Shannon index​ (SMD = −0.08 [95%CI: −0.50, 0.33], *P* = 0.690), also with no heterogeneity (I^2^ = 0.0%, *P* = 0.732) (Figure S2, Table 1). For pre-eclampsia, the pooled analysis demonstrated no significant difference in oral microbial alpha diversity between patients and controls based on the Shannon index (Shannon: SMD = 0.01 [95%CI: −1.63, 1.66], *P* = 0.987, I^2^ = 87.7%) (Figure S3, Table 1). For preterm birth, data from two studies involving 212 participants showed no significant intergroup differences across the Simpson and Chao1 indices (Simpson: SMD = 0.10 [95%CI: −0.52, 0.72], *P* = 0.757, I^2^ = 76.2%; Chao1: SMD = −0.03 [95%CI: −0.51, 0.45], *P* = 0.907, I^2^ = 61.5%) (Figure S4, Table 1).

**Table 1.** Summary of pooled meta-analysis results for alpha-diversity indices.

Disease	Alpha diversity metrics	No. of studies	Total participants (case/control)	Pooled results		Heterogeneity	
				SMD (95% CI)	*P* values	I^2^	*P* values
GDM	Observed richness	4	113/327	−0.29 [−0.55, −0.03]	0.028	23.9%	*P* = 0.268
	ACE index	2	68/94	−0.18 [−1.70, 1.34]	0.814	95%	*P* < 0.001
	Chao1 index	3	94/136	0.04 [−0.73, 0.80]	0.926	87.1%	*P* < 0.001
	Simpson index	3	94/136	−0.22 [−0.90, 0.46]	0.530	84.2%	*P* = 0.002
	Shannon index	6	179/440	−0.08 [−0.26, 0.10]	0.386	0.0%	*P* = 0.588
LBW	Chao1 index	3	42/51	0.46 [0.03, 0.88]	0.035	0.0%	*P* = 0.438
	Shannon index	3	42/51	−0.08 [−0.50, 0.33]	0.690	0.0%	*P* = 0.732
Pre-eclampsia	Shannon index	2	22/35	0.01 [−1.63, 1.66]	0.987	87.7%	*P* = 0.004
Preterm birth	Simpson index	2	91/121	0.10 [−0.52, 0.72]	0.757	76.2%	*P* = 0.040
	Chao1 index	2	91/121	−0.03 [−0.51, 0.45]	0.907	61.5%	*P* = 0.107

Abbreviations: ACE, Abundance-based Coverage Estimator; CI, confidence interval; GDM, gestational diabetes mellitus; LBW, low birth weight; SMD, standardized mean difference.

Note: Total participant numbers represent the cumulative sample size included in each pooled analysis. Individual case and control sample sizes for each study are presented in the corresponding supplementary forest plots.

While some studies could not be incorporated into the meta-analysis due to unavailable raw data, their findings remain pertinent. In a study characterizing the microbiome of South African pregnant women with and without pre-eclampsia, alpha diversity across the gut, vaginal and oral compartments was generally higher in the pre-eclampsia group than in normotensive controls, as assessed by both the Chao1 richness index and the Shannon diversity index. However, no significant intergroup differences were observed in the oral microbiome for either index (Chao1 index: *P* = 1; Shannon index: *P* = 0.715) [29]. Faryal et al. investigated salivary bacterial diversity in relation to adverse pregnancy outcomes (preterm birth, LBW, pre-eclampsia) using culture-dependent and -independent methods [31]. While alpha diversity trends indicated that postpartum females with full-term birth (regardless of oral health status) had higher salivary microbiome diversity than those with preterm birth, these differences were not statistically significant (Mann–Whitney/Kruskal–Wallis test, *P* > 0.05). Their findings also found reduced microbial alpha diversity in participants with oral health complications and preterm birth deliveries compared to those with full-term pregnancies.

#### Association between oral microorganisms during pregnancy and systemic diseases

A meta-analysis was not feasible for the included maternal conditions owing to the paucity of available studies. With respect to depressive symptoms, Agranyoni et al. found no significant differences in alpha diversity indices, including Observed taxa count, Inverse Shannon index and Faith's phylogenetic diversity, between pregnant women with and without depressive symptoms (*P* > 0.05) [22]. Additionally, Alex et al. applied principal component analysis (PCA) and observed higher oral microbial alpha diversity in pregnant women with elevated anxiety or depression scores. In univariate analyzes, increased oral microbial alpha diversity (indicated by higher species richness) was significantly associated with high trait anxiety and depressive symptoms in second-trimester pregnant women (*P* = 0.01 for anxiety; *P* = 0.025 for depression). By contrast, no such associations were detected for other stress and mental health indicators (*P* = 0.485 for life stress; *P* = 0.441 for PTSD), and all these potential correlations failed to be validated in multivariate regression analyzes following adjustment for confounding factors including cigarette smoking and dental morbidities [23]. For pre-existing T2DM in pregnancy, alpha diversity analysis revealed that pregnant women with T2DM exhibited significantly higher Shannon diversity and Species richness in buccal swab samples at late gestation (34–38 weeks) compared to normoglycemic controls (*P* = 0.0264 for Shannon index; *P* = 0.0132 for richness). Besides, a borderline non-significant increase in both indices was observed at early gestation (26–28 weeks of gestation) (*P* = 0.098 for Shannon index; *P* = 0.055 for richness). Notably, no intergroup differences in alpha diversity were detected in saliva rinse samples at either gestational time point [33]. For pregnant women with arterial hypertension, salivary microbial alpha diversity did not differ significantly from normotensive controls in the third trimester (Shannon index: *P* = 0.529). However, Shannon diversity was significantly higher in the hypertensive group than in healthy controls at 6 months postpartum (*P* = 0.013) [25].

### Beta diversity

Compositional differences in microbial communities were assessed using beta diversity metrics, encompassing both dissimilarity calculations (e.g. ANOSIM) and ordination visualizations (e.g. Principal coordinates analysis (PCoA)). Among the 22 studies that reported beta diversity comparisons [22–37,39,40,42–45], substantial methodological heterogeneity, including variations in sample types, distance metrics, statistical frameworks (e.g. PERMANOVA, ANOSIM, analysis of molecular variance [AMOVA], *Adonis2*, mixed-effects models, redundancy analysis [RDA]) and the inconsistent reporting of raw statistics, precluded a formal quantitative meta-analysis. Instead, we conducted a narrative synthesis of beta diversity findings, with full reporting of available exact *P* values, statistical test names, significance thresholds and explicit comparison groups for every included analysis, as detailed in Table S7.

Beta diversity comparisons between GDM-positive pregnant women versus healthy controls yielded highly inconsistent results, with significance dependent on analytical approach. Five studies detected no significant beta diversity differences using PERMANOVA or AMOVA [26,27,32,42,45], with exact *P* values available from three [26,27,42]. Two studies reported significant compositional differences. Specifically, one characterized the microbiota of saliva and dental plaque using Bray–Curtis dissimilarity and PCoA [34], whereas the other compared GDM patients with versus without periodontitis (weighted UniFrac distances, PCoA and heatmap visualization) [36].

Evidence from beta diversity assessments comparing pre-eclamptic patients to normotensive controls remained contradictory. Notably, two investigations observed significant intergroup distinctions: one employing ANOSIM with Bray–Curtis metrics​ (*P* = 0.045) [24], and the other utilizing PERMANOVA with Aitchison distance​ (*P* = 0.015) [32]. Two studies failed to detect significant compositional differences. Specifically, a PERMANOVA analysis revealed non-significant results using both weighted (*P* = 0.354) and unweighted (*P* = 0.185) UniFrac distances [29]. Similarly, a mixed-effect linear model incorporating Aitchison distance also yielded non-significant findings (exact *P* not reported) [28].

In comparisons of preterm versus full-term births, most beta diversity analyzes yielded non-significant results. The exception was a single study of subgingival plaque that employed PERMANOVA with Bray–Curtis and Jaccard distances, reporting significant differences between spontaneous early-term and full-term deliveries; however, the exact *P* value was not provided [43]. Three additional studies found no significant differences via PERMANOVA, with exact *P* values ranging from 0.2346 to 0.908 [30,37]. However, a subgroup analysis by Heo et al. revealed significant distinctions between the two groups after accounting for maternal inflammation [37]. Furthermore, one study utilizing PCoA visualization​ also found no significant separation between these groups [39].

For LBW subgroups, only one study reported significant beta diversity differences between females with caries/gingivitis who had a full-term delivery and those who experienced a preterm LBW delivery via Bray–Curtis distance (Bray‒Curtis = 0.55) [31]. Two other studies were non-significant: one four-group comparison of preterm LBW and periodontitis subgroups (*P* = 0.121) [44], and one comparison of LBW infant delivery females versus normal birth weight infant delivery females (*P* = 0.305) [35]. What's more, Wu et al. indicated that the microbial community structures of pregnant women without periodontal disease who had a healthy delivery seemed to have a tight arrangement compared with those with preterm LBW outcomes, although no statistical differences were found [44].

Among the studies linking oral microbiota to systemic diseases, five reported beta diversity results. Significant beta diversity differences were detected for two systemic conditions: hypothyroidism versus healthy pregnant controls (ANOSIM, *P* < 0.05) [40], and arterial hypertension versus healthy pregnant controls (PERMANOVA, third trimester *P* = 0.02; six months postpartum *P* = 0.03) [25]. Besides, no significant beta diversity differences were observed between pregnant women with pre-existing T2DM versus normoglycemic pregnant controls, regardless of sample type (saliva rinse or swab) [33]. Among mental health disorders, only high PTSD symptom scores versus low PTSD symptom scores showed significant beta diversity differences (PERMANOVA; *P* = 0.037) [23]. No significant differences were found for high versus low stress symptoms (PERMANOVA; *P* = 0.051, borderline non-significant), high versus low anxiety symptoms (PERMANOVA; *P* = 0.429) and high versus low depression symptoms (PERMANOVA; *P* = 0.321). Besides, a separate analysis of pregnant individuals with depressive symptoms versus those without depressive symptoms across trimesters was also non-significant (PERMANOVA; *P* = 0.575) [22].

### Differential relative abundance of microbial taxa

Intergroup statistical tests, including the Wilcoxon rank-sum test (two groups) or Kruskal–Wallis rank test (multiple groups), were used to identify differential taxa, with a subset of studies applying adjusted *P* values (Bonferroni or false discovery rate correction) to mitigate multiple testing errors.

We performed a structured qualitative synthesis of differential taxa reported across eligible studies. Fifteen of the included studies reported quantitative comparisons of oral microbial relative abundance between pregnant women with adverse pregnancy outcomes and healthy controls [22–24,26,27,29,30,32–37,39,40]. Putative within- and between-disorder patterns of relative abundance changes are summarized in Table 2. For GDM, 84 taxa were reported to exhibit​ significant relative abundance differences; for preterm birth, 55 taxa; for pre-eclampsia, 22 taxa; and for LBW, 15 taxa. Consistency in quantitative trends was reported in a subset of these studies: for example, the phylum Bacteroidetes was reported to be increased in relative abundance in GDM and preterm birth groups in select investigations [27,39], while the genera *Granulicatella* and *Streptococcus* were reported to be reduced in GDM and pre-eclampsia groups in limited reports [27,29,34,36]. Similarly, *Aggregatibacter* was enriched, while *Veillonella* and *Leptotrichia* were depleted in GDM [34,36,42]; whereas *Cardiobacterium hominis* and *Campylobacter gracilis* were depleted in preterm birth [30,43]. Recurrent taxa reported in at least two independent studies are summarized in Appendix S4.

**Table 2.** Reported differential abundance of oral microbial taxa in women with adverse pregnancy or birth outcomes compared with controls.

Section 1. Preterm birth (PTB)													
Study		*n* (case/control)	Sampling time point	Sample type		Taxa enriched in cases		Reported quantitative result		Taxa enriched in controls		Reported quantitative result	Statistical method and threshold
Ju Sun Heo et al. (2024) [37]		30/30	Within 3 days postpartum (cesarean delivery)	Subgingival plaque		Order: Actinomycetales		LDA score (log10) Actinomycetales = 4.5		Phylum: Spirochetes Genus: *Treponema, Porphyromonas*		LDA score (log10) Spirochetes = −1.6; *Treponema* = −4.5; *Porphyromonas* = −4.1	LEfSe; Kruskal–Wallis test, *P* < 0.05
Anja Kovanda et al. (2023) [39]		61/91	Upon admission for labor/before planned cesarean section	A pooled saliva, tongue, hard palate and buccal mucosa swab		Phylum: Firmicutes, Bacteroidetes; Genus: *Prevotella*, *Capnocytophaga*, *Veillonella*; Species: *Veillonella massiliensis*		log2FC: (FTB vs PTB) *Prevotella* = −2.2; *Veillonella* = −2.6; *Veillonella massiliensis* = −2.5; Capnocytophaga = −2.2; Firmicutes and Bacteroidetes: exact log2FC NR		Phylum: Proteobacteria Genus: *Leptotrichia*, *Neisseria* spp., *Porphyromonas* spp.; Species: *Haemophilus haemolyticus*		log2FC: (FTB vs PTB) *Haemophilus haemolyticus* = 4.5; *Leptotrichia* = 2.0; *Neisseria* spp. *=* 3.4 ~ 4.4; *Porphyromonas* spp.*=* 2.9 Proteobacteria: exact log2FC NR	DESeq2; Benjamini–Hochberg correction
Jin Kyu Kim et al. (2023) [30]		30/29	24 h before delivery	Saliva		Species: *sp.*HMT 942*, sp.*HMT 080*, bacterium* HMT 505		log2FC: (PTB vs FTB) *sp.*HMT 942 = 2.8; *sp.*HMT 080 = 1.7; *bacterium* HMT 505 = 1.3		Genus: *Saccharibacteria* (TM7) [G-1] spp., *Peptostreptococcaceae* [XI] [G-7] spp., *Kingella* spp., *Corynebacterium* spp.; Species: *Leptotrichia buccalis*, *Lactobacillus gasseri*, *Cardiobacterium valvarum*, *Leptotrichia goodfellowii*, *Saccharibacteria* (TM7) [G-1] *bacterium* HMT 348, *Campylobacter gracilis*, *Prevotella maculosa*, *Treponema* sp. HMT 231, *Prevotella oralis*, *Scardovia wiggsiae*, *Capnocytophaga haemolytica*, *Selenomonas* sp. HMT 442, *Saccharibacteria* (TM7) [G-8] bacterium HMT 955, *Atopobium parvulum*, *Cardiobacterium hominis*, *Olsenella* sp. HMT 807, *Leptotrichia wadei*		log2FC: (PTB vs FTB) *Peptostreptococcaceae* [XI] [G-7] spp. = −1.8; *Kingella* spp. = −2.1; *Corynebacterium* spp. = −2.2; *Leptotrichia buccali*s = −2.7; *Lactobacillus gasseri* = −2.8; *Cardiobacterium valvarum* = −2.7; *Leptotrichia goodfellowii* = −2.1; *Saccharibacteria* (TM7) [G-1] spp. = −2.8; *Campylobacter gracili*s = −2.8; *Prevotella maculosa* = −2.4; *Treponema* sp. HMT 231 = −2.4; *Prevotella oralis* = −2.1; *Scardovia wiggsiae* = −2.2; *Capnocytophaga haemolytica* = −1.7; *Selenomonas* sp. HMT 442 = −2.0; *Saccharibacteria* (TM7) [G-8] bacterium HMT 955 = −1.5; *Atopobium parvulum* = −2.0; *Cardiobacterium hominis* = −2.1; *Olsenella* sp. HMT 807 = −1.7; *Leptotrichia wadei* = −1.4	DESeq2; |log2FC| > 1 and adjust *P* < 0.05
Purnima S. Kumar et al. (2024) [38]		36/45 (PTB + pre-eclampsia vs FTB); 49/45 (PTB vs FTB)	At the time of delivery	Subgingival plaque and saliva		Genus: *Prevotella spp.*, *Fusobacterium spp.*, *Treponema spp.*, *Porphyromonas spp.*, *Streptococcus spp.*, *Actinomyces spp.*		NR		NR		NR	sPLS-DA; LEfSe, LDA > 2
Irene Yang et al. [43]		5/24	8–14 and 24–30 gestational weeks	Subgingival plaque		Species: *Prevotella melaninogenica*		Permutation-based *P*-value: *Prevotella melaninogenica: P* = 0.0435		Species: *Actinomyces israelii*, *Leptotrichia* sp. HMT 392, *Cardiobacterium hominis, Capnocytophaga* sp. HMT 338, *Treponema putidum*, *Bacteroidaceae* [G-1] bacterium HMT 272*, Lautropia mirabilis, Prevotella loescheii*, *Campylobacter gracilis*, *Catonella* sp. HMT 164, *Tannerella s* *p*. HMT 286		Permutation-based *P*-value: *Campylobacter gracilis: P =* 0.025; *Catonella* sp. HMT 164: *P =* 0.0424; *Tannerella* sp. HMT 286: *P =* 0.0452	LDM, FDR = 5%

Section 2. Low birth weight (LBW)													
Study	*n* (case/control)		Sampling time point		Sample type		Taxa enriched in cases		Reported quantitative result		Taxa enriched in controls	Reported quantitative result	Statistical method and threshold
Pei Liu et al. [35]	23/22		10–20 and 28–40 gestational weeks		Saliva		Species: *Bacteroidales* [G-2] bacterium HMT 274, *Filifactor alocis*, *Ruminococcaceae* [G-1] bacterium HMT 075, *Streptococcus* sp. HMT 074, *Streptococcus oralis* subsp*. dentisani* clade 058*, Streptococcus australis*, *Streptococcus salivarius*, *Streptococcus parasanguinis* clade 411, *Granulicatella adiacens*, *Streptococcus cristatus* clade 578, *Gemella sanguinis*, *Leptotrichia buccalis*		*P* for median relative abundance: *Bacteroidales* [G-2] bacterium HMT 274: *P =* 0.036; *Filifactor alocis*: *P* = 0.023; *Ruminococcaceae* [G-1] bacterium HMT 075: *P* = 0.040; *Streptococcus* sp. HMT 074: *P* = 0.005 *Gemella sanguinis: P =* 0.004; *Granulicatella adiacens: P =* 0.002; *Leptotrichia buccalis: P =* 0.046; *Streptococcus australis: P =* 0.001; *Streptococcus cristatus* clade 578: *P* = 0.019; *Streptococcus oralis* subsp*. dentisani* clade 058: *P =* 0.008; *Streptococcus parasanguinis* clade 411: *P* = 0.003; *Streptococcus salivarius: P =* 0.013; *Streptococcus* sp. HMT 074: *P* < 0.001		Phylum: *Saccharibacteria (TM7)*	*P* for median relative abundance: *Saccharibacteria (TM7): P =* 0.015	LEfSe, LDA > 3.5; Wilcoxon Rank Sum Test, *P* < 0.05
Yafei Wu et al. (2021) [44]	19/29		26–28 gestational weeks		Subgingival plaque		None identified		NA		Phylum: Proteobacteria Genus: *Neisseria*, unclassified *Neisseria* spp.	Mean proportion (%): *Neisseria*, *P* = 0.0486; unclassified *Neisseria* spp., *P* < 0.001	Kruskal–Wallis rank-sum test, *P* < 0.05

Section 3. Pre-eclampsia													
Study	*n* (case/control)		Sampling time point		Sample type		Taxa enriched in cases		Reported quantitative result		Taxa enriched in controls	Reported quantitative result	Statistical method and threshold
Marleen M. Kock et al. (2022) [29]	6/5		32–40 gestational weeks		Saliva		Genus: *Prevotella, Haemophilus*; Species: *Prevotella melaninogenica, Neisseria subflava*		Mean relative abundance (%): *Prevotella* 5.16 vs 4.13; *Haemophilus* 13.29 vs 8.40; *Prevotella melaninogenic*a 23.57 vs 17.84; *Neisseria subflava* 6.96 vs 3.93		Genus*: Streptococcus, Granulicatella, Veillonella* Species*: Rothia dentocariosa*	*P* for relative abundance: *Rothia dentocariosa* = 0.02535	Non-parametric Kruskal–Wallis test, *P* < 0.05
Marloes Dekker Nitert et al. (2022) [24]	12/24		36 weeks gestation		Buccal swab		Genus: *Methanosaeta*, *Desulfomicrobium*, *Enterococcus*, *Mycobacterium*, *Thiobacillus*, *Ochrobactrum*		LDA Score: *Methanosaeta* = 3.7; *Desulfomicrobium* = 3.6; *Enterococcus* = 3.5; *Mycobacterium* = 3.5; *Thiobacillus* = 3.4; *Ochrobactrum* = 3.2		Genus: *Veillonella, Prevotella, Roseomonas, Johnsonella, Caulobacter*	LDA Score: *Veillonella* = −4.4; *Prevotella* = −4.2; *Roseomonas* = −3.8; *Johnsonella* = −3.8; *Caulobacter* = −3.9	LEfSe, LDA ≥ 2.0
Purnima S. Kumar et al. (2024) [38]	36/45 (PTB + pre-eclampsia vs FTB)		At the time of delivery		Subgingival plaque and saliva		Genus: *Prevotella* spp., *Fusobacterium* spp., *Treponema* spp., *Porphyromonas* spp.		NR		NR	NR	sPLS-DA; LEfSe, LDA > 2

Section 4. Gestational Diabetes Mellitus (GDM)													
Study	*n* (case/control)		Sampling time point		Sample type		Taxa enriched in cases		Reported quantitative result		Taxa enriched in controls	Reported quantitative result	Statistical method and threshold
Jizhi Zhao et al. (2021) [45]	total = 69		20–28 gestational weeks		Subgingival plaque, saliva		Order: Unidentified Flavobacteriales, unidentified Bacillales; Family: Christensenellaceae, Ruminococcaceae, Enterobacteriaceae Species: *Bacteroides eggerthii*		LDA Score: Unidentified Flavobacteriales *=* 3.2; unidentified Bacillales = 3.2; Christensenellaceae = 2.7; Ruminococcaceae = 4.1; Enterobacteriaceae = 3.5; *Bacteroides eggerthii =* 2.1		Family: Saprospiraceae, Microscillaceae, Rhodocyclaceae, Akkermansiaceae	LDA Score: Saprospiraceae = −2.2; Microscillaceae = −2.5; Rhodocyclaceae = −2.8; Akkermansiaceae = −2.4	LEfSe, LDA > 2.0
Yajuan Xu et al. [42]	27/30		Third trimester of pregnancy		Saliva		Order: Bifidobacteriales Family: Bifidobacteriaceae Genus: *Selenomonas*, *Bifidobacterium*		LDA Score: Bifidobacteriales = 2.7; Bifidobacteriaceae *=* 2.7; *Selenomonas =* 3.1; *Bifidobacterium =* 2.7		Phylum: Fusobacteriota Class: Fusobacteriia Order: Fusobacteriales Family: Leptotrichiaceae, Leuconostocaceae; Genus: *Leuconostoc, Lachnoanaerobaculum,* *Leptotrichia*	LDA Score: Fusobacteriota = −4.2; Fusobacteriia = −4.2; Fusobacteriales = − 4.2; Leptotrichiaceae = −4.2; Leuconostocaceae = −3.9; *Leuconostoc* = −3.9; *Lachnoanaerobaculum =* −2.7; *Leptotrichia =* −4.2	LEfSe
Mie K. W. Crusel et al. [27]	50/160		27–33 gestational weeks		Saliva		Phylum: Bacteroidetes Class: Bacteroidia, Clostridia; Order: Bacteroidales, Clostridiales; Family: Prevotellaceae, Veillonellaceae; Genus: *Prevotella*		log2FC: *Prevotella* (OTU_393) *=* 1.88; Veillonellaceae (OTU_623) = 2.8		Phylum: Proteobacteria Class: Betaproteobacteria, Gammaproteobacteria, Bacilli; Order: Neisseriales, Pasteurellales, Lactobacillales; Family: Neisseriaceae, Pasteurellaceae, Streptococcaceae; Genus*: Neisseria*, *Actinobacillus*, *Streptococcus*; Species: *Actinobacillus parahaemolyticus*	log2FC: *Neisseria* (OTU_387) *= −*1.5; *Actinobacillus parahaemolyticus* (OTU_196) *= −*2.1; *Streptococcus* (OTU_279) *= −*0.79	DESeq2 negative binomial regression; FDR < 10% (Benjamini-Hochberg)​
Fangqing Zhao et al. (2018) [41]	total = 346		1–2 days before delivery		Saliva		Phylum: Proteobacteria		Relative abundance:(GDM VS control) Proteobacteria: 15–55% VS 12–52%		Phylum: Firmicutes	Relative abundance:(GDM VS control) Firmicutes: 18–37% VS 20–40%	PLS-DA; Mann –Whitney U test with Benjamini–Hochberg FDR correction, *P* < 0.05
Jinfeng Wang et al. (2020) [34]	41/64		Third trimester of pregnancy		Saliva and dental plaque		Order: Neisseriales, Cardiobacteriales; Family: Leptotrichiaceae, Neisseriaceae, Pseudonocardiaceae, Weeksellaceae, Comamonadaceae, Cardiobacteriaceae; Genus: *Lautropia*, *Neisseria*, *Kocuria*, *Paludibacter*, *Capnocytophaga*, *Flavobacterium*, *Mycoplana*, *Cardiobacterium*, *Citrobacter*, *Aggregatibacter*		LDA score (log10): Neisseriales = 4.8; Cardiobacteriales *=* 3.0; Leptotrichiaceae = 4.2; Neisseriaceae = 3.0; Pseudonocardiaceae = 3.5; Comamonadaceae = 3.0; *Lautropia =* 4.5; *Neisseria* = 4.3; *Kocuria =* 3.0; *Paludibacter =* 3.0; *Capnocytophaga =* 4.5; *Mycoplana =* 3.0; *Cardiobacterium =* 3.0; *Citrobacter =* 3.3; *Aggregatibacter =* 3.1		Family: F16 Genus: *Selenomonas, Leptotrichia,* *Streptococcus, Veillonella*	LDA score (log10): F16 = *−*3.0; *Selenomonas* = *−*3.6; *Leptotrichia = −*4.2; *Streptococcus = −*4.8; *Veillonella = −*4.2	LEfSe, LDA > 3.0
Xiu-Min Jiang et al. (2025) [36]	40/40		Third trimester of pregnancy		Saliva		Phylum: Bacteroidota, Fusobacteriota, Spirochaetota, Synergistota, Chloroflexi; Genus: *Fusobacterium*, *Treponema*, *Aggregatibacter*, *Filifactor*, *Porphyromonas*		NR		Phylum: Firmicutes, Actinobacteriota, Patescibacteria; Genus: *Veillonella*, *Rothia*, *Leptotrichia*, *TM7x*, *Actinomyces*, *Granulicatella*, *Oribacterium*, *Corynebacterium*	NR	Mann–Whitney U test with FDR correction, *P* < 0.05

Abbreviations: DESeq2, Differential Expression Analysis for Sequence count data; FDR, False discovery rate; FTB, Full-term birth; GDM, Gestational diabetes mellitus; LBW, Low birth weight; LDA, Linear discriminant analysis; LDM, Linear decomposition model; LEfSe, Linear discriminant analysis effect size; Log2FC, Log2 fold change; NA, Not applicable; NR, Quantitative effect size not reported by the original study; OTU, Operational taxonomic units; PLS-DA, partial least squares discriminant analysis; PTB: Preterm birth; sPLS-DA, Sparse partial least squares discriminant analysis.

Five of the included studies reported quantitative comparisons of oral microbial relative abundance between pregnant women with systemic diseases and healthy controls, identifying taxa with significant intergroup differences [22,23,25,33,40]. The number of taxa reported to exhibit​ significant relative abundance differences was 30 for hypothyroidism, 12 for mental health symptoms (anxiety, depression, PTSD), 10 for arterial hypertension and 9 for T2DM. In a subset of these studies, consistency in quantitative trends was reported for select taxa; the phylum Proteobacteria was reported to be increased in relative abundance in both mental health disorders and hypothyroidism in limited investigations [23,40]; the genus *Dialister* was enriched in both mental health disorders and arterial hypertension in limited studies [23,25]; the genera *Streptococcus*, *Neisseria*, *Fusobacterium* and *Capnocytophaga* were reported to be reduced in maternal mental disorders and arterial hypertension in select reports [22,25]; *Haemophilus* was depleted in both T2DM and arterial hypertension in individual studies [25,33]; and *Rothia* showed reduced relative abundance in both hypothyroidism and arterial hypertension in limited datasets [25,40]. No disorder-specific shifts were evident in the remaining taxa (Table 3).

**Table 3.** Different relative abundance on taxa levels between pregnant women with systemic diseases and controls.

Study	Disease	*n* (case/control)	Sampling time point	Sample type	Taxa enriched in cases	Taxa enriched in controls	Statistical method and significance threshold
Sophie M. Leech et al. [33]	T2DM	11/28	26–28 weeks of gestation; 34–38 weeks of gestation;	Buccal swab and Saliva rinse	Family: *Flavobacteriaceae* Genus: *Capnocytophaga* Species: *Capnocytophaga gingivalis, Capnocytophaga leadbetteri, Neisseria elongata*	Phylum: Proteobacteria Order: Pasteurellales Family: *Pasteurellaceae* Genus: *Haemophilus*	A consensus approach for differential abundance analysis; FDR, *P *< 0.05
Joseph S Lonstein et al. (2024)[23]	Maternal mental health (stress, anxiety, depression, PTSD)	224	Second trimester (~17 ± 2 weeks gestation)	Saliva	Phylum: Proteobacteria, Bacteroidetes, Firmicutes, Spirochetes, Genus: *Dialister, Eikenella*	NA	LEfSe; MaAsLin2, *P* < 0.05
Sarven Sabunciyan et al. (2025)[22]	Maternal mental health (depressive symptoms during pregnancy)	46/327	First-third trimester	Saliva	NA	Genus: *Neisseria, Fusobacterium, Capnocytophaga*, *Streptococcus*	ZINB model; DESeq2; *P* (padj) ≤ 0.10
Yajuan Xu et al. [42]	Hypothyroidism	30/31	37–40 gestational weeks	Saliva	Phylum: Proteobacteria Class: Gammaproteobacteria, Betaproteobacteria Order: Pasteurellales, Neisseriales, Family: *Pasteurellaceae, Paraprevotellaceae, Neisseriaceae,* Genus: *Prevotella, Neisseria, Haemophilus,* Species: *Haemophilus_influenzae, Haemophilus_parainfluenzae, Neisseria_subflava*	Phylum: Actinobacteria, Fusobacteria, Firmicutes Class: Bacilli, Fusobacteriia Order: Fusobacteriales, Actinomycetales, Gemellales Family: *Micrococcaceae, Gemellaceae, Carnobacteriaceae* *Leptotrichiaceae* Genus: *Leptotrichia, Rothia, Granulicatella* Species: *Rothia_mucilaginosa*	LEfSe, LDA ≥ 4.0
Maria João Azevedo et al. [25]	Arterial hypertension	10/10	Third trimester of pregnancy and 24–28 weeks after delivery	Saliva	Genus*: Dialister*	Genus*: Neisseria, Fusobacterium, Rothia, Capnocytophaga, Lautropia, Gemella, Bergeyella, Streptococcus, Haemophilus*	ALDEx2; Benjamini–Hochberg, *P *< 0.05

Abbreviations: ALDEx2, ANOVA-Like Differential Gene Expression Analysis; FDR, False Discovery Rate; LEfSe, Linear discriminant analysis Effect Size; LDA, Linear Discriminant Analysis; MaAsLin2, Microbiome Multivariable Associations with Linear Models 2; T2DM, Type 2 Diabetes Mellitus; PTSD, Post-Traumatic Stress Disorder; ZINB: Zero-inflated negative binomial.

## Discussion

The present study systematically evaluated alterations in the oral microbiota of pregnant women by comparing microbial profiles between those with pregnancy complications or systemic diseases and healthy controls. While a recent meta-analysis by Rodríguez-Lozano et al. examined the association between oral dysbiosis and pregnancy-related complications, its primary focus was quantifying differences in alpha diversity (Shannon index) between preterm and term birth groups, without exploring beta diversity or taxon-specific abundance changes [46]. To our knowledge, this review is among the first to comprehensively synthesize both the composition and diversity of the oral microbiome across a range of pregnancy complications and maternal systemic diseases. Key findings are summarized as follows. We observed a significant decrease in Observed richness in GDM (SMD = –0.29 [–0.55, –0.03]) and a significant increase in Chao1 in LBW (SMD = 0.46 [0.03, 0.88]), indicating divergent alpha diversity alterations across complications. In contrast, no significant differences in alpha diversity were detected for pre-eclampsia or preterm birth. Evidence linking oral microbiome beta diversity to adverse pregnancy outcomes remained highly heterogeneous and inconclusive, largely owing to marked methodological disparities across studies. Furthermore, most reported relative abundance changes were unique to individual studies; although limited consistencies emerged, such as increased Bacteroidetes in GDM and preterm birth, and reduced *Granulicatella* and *Streptococcus* in GDM and pre-eclampsia, these patterns lacked independent replication and should be interpreted as preliminary.

Alpha diversity indices are widely used in microbiome research to characterize microbial richness and community diversity within samples. Alterations in alpha diversity have been associated with microbial dysbiosis in various disease conditions, including pregnancy complications [47]. Animal studies have shown that reduced microbial diversity is associated with metabolic dysfunction and enhanced inflammatory responses, supporting the hypothesis that microbial dysbiosis may contribute to adverse pregnancy outcomes [48]. However, our meta-analysis identified significantly lower Observed richness in women with GDM and significantly higher Chao1 values in LBW pregnancies, whereas no significant differences were observed in the ACE, Simpson, or Shannon indices. These findings suggest that the association between oral microbial diversity and pregnancy-related conditions is outcome-specific rather than reflecting a consistent pattern across different complications. The reduced microbial richness observed in GDM may reflect subtle alterations in microbial composition associated with metabolic dysregulation, whereas the elevated Chao1 index in LBW pregnancies may indicate microbial ecosystem instability resulting from the expansion of low-abundance or opportunistic taxa rather than a genuinely healthier microbiota. The findings for LBW should be interpreted with caution, as they were derived from a limited number of studies and were not consistently supported by other diversity indices. Similarly, the lack of significant alpha diversity differences in pre-eclampsia and preterm birth highlights the need for larger, well-designed prospective studies to clarify these associations.

Recent interventional studies provide important contextual support for the observational findings of the present review. Two randomized controlled trials from the OP-PE program in Senegal evaluated the effect of daily calibrated interdental brushing during pregnancy. Bourgeois et al. conducted a cluster randomized controlled trial across six antenatal care facilities in Senegal, enrolling nulliparous pregnant women and evaluating the effect of daily interdental cleaning with standardized interdental brushes initiated at 12 weeks of gestation on a composite adverse obstetric outcome, comprising preterm birth, low birth weight, small for gestational age and pre-eclampsia. Although the intervention did not significantly reduce the composite obstetrical endpoint, it was safe and feasible, with high participant adherence throughout the trial [49]. A secondary analysis of the same trial by Carrouel et al. demonstrated that the interdental brushing intervention significantly reduced gingival bleeding throughout pregnancy in women with an intact periodontium, indicating that pregnancy-associated gingivitis can be improved through simple preventive strategies [50]. A parallel microbiological sub-study by Carrouel et al. further demonstrated that daily interdental brushing substantially reduced the interdental bacterial burden, including a 36.6% reduction in total bacterial load and a 92.1% reduction in *Treponema denticola* abundance by the eighth month of pregnancy, whereas both increased in the control group. Reductions in *Porphyromonas gingivalis* and *Tannerella forsythia* were also observed, though these did not reach statistical significance [51]. Collectively, these three trials suggest that pregnancy-associated oral dysbiosis, including the expansion of red-complex periodontal pathogens previously associated with adverse pregnancy outcomes, may be amenable to targeted interdental prophylaxis. Although these trials do not alter the quantitative estimates derived from our meta-analysis, they complement the observational evidence by strengthening the biological plausibility of the associations between the oral microbiome and adverse pregnancy outcomes. They also highlight the need for adequately powered randomized controlled trials to determine whether microbiome-targeted oral interventions can ultimately improve maternal and neonatal outcomes.

Beta diversity reflects compositional dissimilarity between microbial communities and is typically evaluated via ordination approaches such as PCoA and statistical testing such as PERMANOVA. Substantial methodological heterogeneity across eligible studies, including variable sequencing depths, distinct analytical pipelines and inconsistent reporting of test statistics, precluded formal quantitative meta-analysis of beta diversity. We therefore synthesized these findings narratively, with cautious interpretation given the limited comparability of raw data across investigations. Furthermore, conventional beta diversity analyzes typically rely on pairwise group comparisons, potentially overlooking the complexity of pregnancy-associated microbial community structure. Future studies should adopt more refined analytical frameworks beyond conventional two-group comparisons to capture microbiota variation at higher resolution and evaluate the clinical applicability of beta diversity as a diagnostic or prognostic biomarker.

Microbial findings across pregnancy-related and systemic conditions were largely exploratory and exhibited substantial heterogeneity. To facilitate evidence synthesis, we identified several recurrent taxonomic signatures across independent studies. Notably, reduced abundance of *Leptotrichia*-related taxa was repeatedly observed in women with GDM and preterm birth, while *Veillonella* depletion was reported across studies of GDM and pre-eclampsia. Additional shared findings included increased Bacteroidetes in GDM and preterm birth, reduced *Granulicatella* and *Streptococcus* in GDM and pre-eclampsia, and alterations in Proteobacteria, *Dialister*, *Haemophilus* and *Rothia* across studies of mental health disorders, hypothyroidism and hypertension. Several of these recurrently altered taxa have plausible biological relevance. Members of the genus *Leptotrichia* are common constituents of the healthy oral microbiota; however, alterations in their abundance have been reported in oral inflammatory conditions, and *Leptotrichia* species have been implicated in modulating host cytokine responses, including IL-1β, IL-6, IL-8 and IL-10, suggesting that their depletion may reflect disturbances in oral microbial homeostasis during pregnancy complications [52,53]. Similarly, *Veillonella* species contribute to oral biofilm homeostasis through lactate metabolism and microbial cross-feeding [54]; their reduced abundance may indicate altered oral ecology in women with GDM and pre-eclampsia. *Streptococcus* and *Granulicatella* are integral constituents of the healthy oral microbiota that limit pathogen colonization via competitive exclusion and antimicrobial peptide secretion while modulating local immune responses [55]. Likewise, *Rothia* has been associated with short-chain fatty acid production and potential anti-inflammatory and metabolic benefits [40], and its depletion may reflect broader disruption of microbial homeostasis. Elevated Bacteroidetes abundance has also been associated with excessive gestational weight gain, a well-established risk factor for GDM [56]. This raises the possibility that increased Bacteroidetes abundance may also be a feature of GDM pregnancies, though direct evidence is currently lacking and further investigation is needed to confirm this hypothesis. Interpretation of these taxonomic findings should remain cautious, as most differentially abundant taxa have been reported by only a few studies and lack independent replication.

Considerable heterogeneity was observed across included studies, attributable primarily to methodological and clinical variability. Marked differences in oral microbiota composition across sampling sites, including saliva, subgingival plaque, buccal mucosa and mixed oral samples, preclude direct comparisons between studies. As a key determinant of oral microbiota composition, periodontal status was inconsistently assessed, reported and adjusted for across studies. Differences in sequencing platforms, targeted 16S rRNA hypervariable regions, sequencing depth, taxonomic resolution, bioinformatic pipelines, taxonomic annotation and differential abundance analysis methods further contributed to methodological heterogeneity across studies. Furthermore, adjustment for key confounding variables was inconsistent across studies, including maternal age, body mass index, smoking status, antibiotic use, oral hygiene practices, dietary habits, gestational age at sampling and condition-specific clinical characteristics such as glycemic control in GDM studies. Taken together, these methodological and clinical differences likely underlie the inconsistent microbiota profiles reported across studies and limit the feasibility of robust quantitative synthesis at the taxon level. Future studies should implement standardized sampling, sequencing, bioinformatic and reporting practices to better define host-microbiota interactions underlying adverse pregnancy outcomes.

This meta-analysis reinforces the observed associations between oral microbial communities and adverse pregnancy outcomes and highlights the importance of methodological standardization and transparent reporting for future microbiome research. To account for between-study heterogeneity, pooled effect estimates were generated using a random-effects model, which incorporates both within-study and between-study variance to yield more conservative and generalizable estimates.

Nevertheless, this study has several limitations worth noting. First, substantial heterogeneity was observed among the included studies, largely attributable to differences in participant demographic characteristics, sample collection time points, sequencing platforms and bioinformatics analysis pipelines. Due to the limited number of studies meeting the inclusion criteria, this review was unable to reliably quantify or further explore between-study heterogeneity through subgroup analyzes or meta-regression. Second, the relatively small cumulative sample size reduced statistical power and increased the susceptibility of the pooled estimates to random error and potential publication bias. Accordingly, non-significant findings from the primary studies, as well as null or borderline pooled estimates from the present meta-analysis, should be interpreted with caution because limited statistical power may have masked true associations with small but biologically meaningful effects. Third, this review was unable to perform taxon-level quantitative meta-analysis of microbial relative abundance. Although several independent studies consistently reported associations between certain genera and the same pregnancy complications, reliable quantitative synthesis was not feasible due to substantial variation in reporting practices and methodological approaches across studies. This was primarily due to inconsistent effect metrics employed across studies, such as LDA scores from LEfSe, log2 fold changes, relative abundance values, or *P* values alone, the absence of variability estimates such as standard errors or variances in most studies, and considerable heterogeneity in sample sources, taxonomic resolution, sequencing platforms and bioinformatics pipelines. Future studies should adopt standardized reporting practices, including mean relative abundance with accompanying variance measures, to enable taxon-level meta-analytic synthesis. In light of these limitations, all associations reported in the present study should be regarded as exploratory and hypothesis-generating rather than conclusive, and independent replication in larger, well-designed prospective cohorts is warranted.

## Conclusions

Alpha‑diversity alterations diverged across complications: decreased Observed richness in GDM and increased Chao1 in LBW, but no significant changes in pre‑eclampsia or preterm birth. Similarly, beta diversity analyzes revealed inconsistent compositional shifts, with statistically significant differences detected only in specific subgroups. Qualitative synthesis of taxon-level data identified sparse but recurring patterns. However, methodological variability and incomplete reporting across the included studies limit the robustness of these findings, therefore requiring cautious interpretation and highlighting the need for standardized, high-quality research in this emerging field.

## Supplementary Material

Figure S2.tif Figure S2.tif

Appendix S4.docx Appendix S4.docx

Supplementary Table 1.docx Supplementary Table 1.docx

Figure S3.tif Figure S3.tif

Supplementary Table 4.docx Supplementary Table 4.docx

Appendix S2.docx Appendix S2.docx

Appendix S3.docx Appendix S3.docx

Supplementary Table 2.docx Supplementary Table 2.docx

Supplementary Table 3.docx Supplementary Table 3.docx

Figure S1.tif Figure S1.tif

Supplementary Table 7.docx Supplementary Table 7.docx

Supplementary Table 6.docx Supplementary Table 6.docx

PRISMA checklist.docx PRISMA checklist.docx

Supplementary Table 5.docx Supplementary Table 5.docx

Appendix S1.docx Appendix S1.docx

Figure S4.tif

Supplementary Material Supplementary_figure_caption.docx

## Data Availability

No new datasets were generated or analyzed during the current study.
