# Supplementary material for: The oral microbiome in relation to systemic maternal conditions and pregnancy complications: a systematic review and meta-analysis
Source: J Oral Microbiol. 2026 Aug 26;18(1):2717774. doi: 10.1080/20002297.2026.2717774 (PMC13520856; doi:10.1080/20002297.2026.2717774)
Supplement: Supplementary file 1 [file ZJOM_A_2717774_SM2851.docx]

**Appendix S4. Recurrently reported taxa associated with adverse pregnancy/birth outcomes.**

| **Outcome** | **Recurrently enriched taxa (≥2 studies)** | **Recurrently depleted taxa (≥2 studies)** |
| --- | --- | --- |
| Preterm birth | None identified | *Leptotrichia spp.*, *Cardiobacterium hominis,* *Campylobacter gracilis,*  *Neisseria spp.* |
| Low birth weight | None identified | None identified |
| Pre-eclampsia | None identified | *Veillonella* |
| Gestational diabetes mellitus | *Aggregatibacter* | *Veillonella, Leptotrichia*. |
