# Supplementary material for: The oral microbiome in relation to systemic maternal conditions and pregnancy complications: a systematic review and meta-analysis
Source: J Oral Microbiol. 2026 Aug 26;18(1):2717774. doi: 10.1080/20002297.2026.2717774 (PMC13520856; doi:10.1080/20002297.2026.2717774)
Supplement: Supplementary file 2 [file ZJOM_A_2717774_SM2852.docx]

**Table S1. Association between oral microorganisms during pregnancy and adverse birth outcomes.**

| **Author(year)** | **Study type** | **Outcomes** | **Microbial detection methods** | **Sample source** | **Sample collection time** | **Amplified region** | **Sequencing platform** | **Raw data availability** |
| --- | --- | --- | --- | --- | --- | --- | --- | --- |
| Irene Yang et al. (2022) | Longitudinal descriptive study | Preterm birth | 16S rRNA Sequencing | Subgingival plaque | 8–14 and 24–30 gestational weeks | (v3-v4) | Illumina MiSeq | NCBI: PRJNA811442 |
| Yafei Wu et al. (2021) | Prospective study | LBW | 16S rRNA Sequencing | Subgingival plaque | 26–28 gestational weeks | (v3-v4) | Illumina MiSeq | SRA:  PRJNA763000 |
| Ju Sun Heo et al. (2024) | Cross-sectional study | Preterm birth | 16S rDNA sequencing | Subgingival plaque | Within 3 days postpartum (caesarean delivery) | (v3-v4) | Illumina MiSeq | NR |
| Anja Kovanda et al. (2023) | Case control study | Preterm birth | 16S rRNA sequencing | A pooled saliva, tongue, hard palate, and buccal mucosa swab | Upon admission for labor/before planned caesarean section | (v3-v4) | Illumina MiSeq | https://www.ncbi.nlm.nih.gov/,PRJNA913792 |
| Pei Liu et al. (2024) | Nested case-control study | LBW | 16S rRNA sequencing | Saliva | 10–20 and 28–40 gestational weeks | (v3-v4) | Illumina MiSeq | NCBI |
| Jin Kyu Kim et al. (2023) | Prospective study | Preterm birth | 16S rRNA Sequencing | Saliva | 24 hours before delivery | (v3-v4) | Illumina MiSeq | SRA:  PRJNA985119 |
| Rani Faryal et al. (2021)* | Cross-sectional study | Preterm birth, LBW, pre-eclampsia | 16S rRNA sequencing | Saliva | After first day of delivery | (v4) | Illumina MiSeq | SRA: PRJNA505914 |
| Rebecca Koerner et al. (2022)* | Case control study | Preterm birth, GDM, pre-eclampsia | 16S rRNA sequencing | Saliva | 17–19 gestational weeks, 25–27 gestational weeks, and 32–34 gestational weeks | (v4) | Illumina MiSeq | NR |
| Purnima S. Kumar et al. (2024)* | Case control study | Preterm birth, pre-eclampsia | whole-genome shotgun sequencing | Subgingival plaque and saliva | At the time of delivery | NA | Illumina HiSeq4000 | SRA of the NCBI database: PRJNA934596 |

*Abbreviations:* *LBW, Low birth weight; GDM, Gestational diabetes mellitus; NA, Not applicable; NCBI, National Center for Biotechnology Information; NR, not reported;* *SRA, Sequence Read Archive; rRNA, ribosomal RNA.*

**Footnote:**

*** Rani Faryal et al. (2021) is listed in both Table S1 and Table S2 as it examined both preterm birth, low birth weight (birth outcome) and pre-eclampsia (pregnancy outcomes).**

***Rebecca Koerner et al. (2023) is listed in both Table S1 and Table S2 as it examined both preterm birth (birth outcome), pre-eclampsia and GDM (pregnancy outcomes).**

***Purnima S. Kumar et al. (2024) is listed in both TableS1 and Table S2 as the study examined both preterm birth (birth outcome) and pre-eclampsia (pregnancy outcome).**
