# Supplementary material for: The oral microbiome in relation to systemic maternal conditions and pregnancy complications: a systematic review and meta-analysis
Source: J Oral Microbiol. 2026 Aug 26;18(1):2717774. doi: 10.1080/20002297.2026.2717774 (PMC13520856; doi:10.1080/20002297.2026.2717774)
Supplement: Supplementary file 3 [file ZJOM_A_2717774_SM2854.docx]

**Table S4. Quality assessment of the included case‒control studies.**

| **Reference** | **Disease** | **Selection** | | | | **Comparability** | | **Exposure** | | | **Total score** |
| --- | --- | --- | --- | --- | --- | --- | --- | --- | --- | --- | --- |
|  |  | **Is the case definition adequate?** | **Representativeness of the cases** | **Selection of Controls** | **Definition of Controls** | **Study controls for most important factor** | **Study controls for any additional factor** | **Ascertainment of exposure** | **Same method of ascertainment for cases and controls** | **Non-Response rate** |  |
| **Jinfeng Wang et al. (2020)** | **GDM** | **+** | **-** | **+** | **+** | **+** | **+** | **+** | **+** | **-** | **7** |
| **Yajuan Xu et al. (2020)** | **Hypothyroidism** | **+** | **-** | **+** | **+** | **+** | **+** | **+** | **+** | **-** | **7** |
| **Marloes Dekker Nitert et al. (2022)** | **Pre-eclampsia** | **+** | **-** | **+** | **+** | **+** | **-** | **+** | **+** | **-** | **6** |
| **Zoe Brookes et al. (2025)** | **Pre-eclampsia** | **+** | **-** | **+** | **+** | **+** | **+** | **+** | **+** | **-** | **7** |
| **Anja Kovanda et al. (2023)** | **Preterm birth** | **+** | **-** | **+** | **+** | **+** | **-** | **+** | **+** | **-** | **6** |
| **Sophie M. Leech et al. (2025)** | **T2DM** | **+** | **+** | **+** | **+** | **+** | **-** | **+** | **+** | **-** | **7** |
| **Xiu-Min Jiang et al. (2025)** | **GDM** | **+** | **-** | **+** | **+** | **+** | **-** | **+** | **+** | **-** | **6** |
| **Rebecca Koerner et al. (2022)** | **Pre-eclampsia, preterm birth, GDM** | **+** | **-** | **+** | **+** | **+** | **-** | **+** | **+** | **-** | **6** |
| **Purnima S. Kumar et al. (2024)** | **Pre-eclampsia, preterm birth** | **+** | **-** | **+** | **+** | **+** | **-** | **+** | **+** | **-** | **6** |
| **Yajuan Xu et al (2019)** | **GDM** | **+** | **-** | **+** | **+** | **+** | **-** | **+** | **+** | **-** | **6** |
| **Pei Liu et al (2024)** | **LBW** | **+** | **-** | **+** | **+** | **+** | **-** | **+** | **+** | **-** | **6** |
| **Fangqing Zhao et al (2018)** | **GDM** | **+** | **-** | **+** | **+** | **+** | **-** | **+** | **+** | **-** | **6** |

Scale case control studies: Selection: 1) Is the case definition adequate? a) yes, with independent validation*; 2) representativeness of the cases: a) consecutive or obviously representative series of cases*; 3) Selection of controls: a) community controls*; 4) Definition of controls a) no history of disease (endpoint) *; Comparability: 1) Study controls for most important factor a) yes*, 2) Study controls for any additional factor a) yes*; Exposure: 1) Ascertainment of exposure*: a) secure record), b) structured interview where blind to case/control status; 2) Same method of ascertainment for cases and controls a) yes*; 3) Non-Response rate a) same rate for both groups* A study can be awarded a maximum of one star for each numbered item within the Selection, Comparability and Exposure categories.

**Selection**: The authors provide a description of where the case and control population are taken from the same location and matched in their characteristics.

**Comparability:** Cases and controls were evaluated using the same criteria and for the same duration.

**Outcome:** The case and control groups were exposed to the same analyzes for the same period of time using the same methodology.

*Abbreviations:* *GDM, Gestational diabetes mellitus; LBW: Low birth weight; T2DM: Type 2 Diabetes Mellitus.*
