# Supplementary material for: The oral microbiome in relation to systemic maternal conditions and pregnancy complications: a systematic review and meta-analysis
Source: J Oral Microbiol. 2026 Aug 26;18(1):2717774. doi: 10.1080/20002297.2026.2717774 (PMC13520856; doi:10.1080/20002297.2026.2717774)
Supplement: Supplementary file 4 [file ZJOM_A_2717774_SM2855.docx]

| **Appendix S2. Assessment criteria for microbiome-specific methodological risk of bias.** | | | |
| --- | --- | --- | --- |
| **Domain** | **Low risk of bias** | **Unclear risk of bias** | **High risk of bias** |
| **Microbial measurement reliability** | Clear sampling protocol; validated DNA extraction kit; reported 16S rRNA hypervariable region and primers | Incomplete description of sampling or library preparation; no information on primer or kit validation | No key sampling details; non‑validated DNA extraction method; unclear sequencing target region |
| **Sequencing and batch effect control** | Reported sequencing platform and QC; batch effect controlled by randomization or correction; normalized sequencing depth | Sequencing platform reported but no QC or batch correction stated; no rarefaction or depth normalization | No sequencing or QC details; obvious uncorrected batch effects; highly uneven sequencing depth without adjustment |
| **Bioinformatic pipeline consistency** | Clear pipeline (e.g., QIIME2/DADA2); specified reference database and version; raw data deposited in public repository | Pipeline mentioned but key parameters unclear; database not specified | Vague or no bioinformatic description; no taxonomic database reported; no raw data provided |
| **Statistical analysis appropriateness** | Proper normalization (e.g., CLR/TSS); appropriate statistical tests; adjustment for multiple comparisons | Normalization or statistical method mentioned but not detailed; no multiple testing correction reported | No data normalization; inappropriate statistical tests; no adjustment for multiple comparisons |
