# Supplementary material for: The oral microbiome in relation to systemic maternal conditions and pregnancy complications: a systematic review and meta-analysis
Source: J Oral Microbiol. 2026 Aug 26;18(1):2717774. doi: 10.1080/20002297.2026.2717774 (PMC13520856; doi:10.1080/20002297.2026.2717774)
Supplement: Supplementary file 5 [file ZJOM_A_2717774_SM2856.docx]

**Appendix S3.Key justifications for risk of bias level.**

| Disease | Study ID | Key justifications |
| --- | --- | --- |
| GDM | Jinfeng Wang et al. (2020) | The study had standardized methodological design, complete experimental details, reproducible analysis pipelines and statistical methods suitable for microbiome data, meeting the high-quality methodological requirements for microbiome research. |
| GDM | Xiu-Min Jiang et al. (2025) | The three core technical domains all achieved low risk of bias, with rigorous, standardized, and fully documented experimental design and execution. The only unclear risk is limited to the statistical analysis domain (lack of multiple comparison adjustment) and does not undermine the fundamental reliability of the study’s experimental data and core findings. |
| GDM | Jizhi Zhao et al. (2021) | The study’s microbial research methods have an unclear overall risk of bias, with only the Bioinformatic pipeline consistency domain achieving a low risk rating (rigorous, reproducible, and fully documented analysis workflow). The other three core domains were rated unclear risk due to incomplete methodological reporting and key omissions (e.g., no batch effect control measures, lack of multiple comparison correction). |
| GDM | Silvia Daher et al. (2018) | The study achieved **low risk of bias** in the three core technical domains. The only unclear risk is limited to the statistical analysis appropriateness domain, arising from incomplete reporting of formal multiple comparison correction methods and normalization strategy justification (rather than inappropriate statistical practices). This omission does not undermine the fundamental reliability of the study’s experimental design, data generation, or core findings. |
| GDM | Mie K. W. Crusel et al. (2020) | The study achieved low risk of bias in the three core technical domains. The only unclear risk is limited to the statistical analysis domain, arising from incomplete justification of the normalization strategy (rarefaction) and ambiguous reporting of multiple comparison adjustment for longitudinal alpha diversity analyses. |
| GDM | Yajuan Xu et al. (2019) | The study achieved low risk of bias in two core domains: microbial measurement reliability (standardized sampling, validated extraction, clear sequencing targets) and bioinformatic pipeline consistency (transparent workflow, public data deposition). However, the other two critical domains (sequencing and batch effect control, statistical analysis appropriateness) were rated unclear risk due to key methodological omissions (e.g., lack of QC metrics, unaddressed batch effects, incomplete normalization justification). The main limitations lie in incomplete methodological reporting rather than flawed experimental design, which limits full assessment of reproducibility and rigor. |
| GDM | Fangqing Zhao et al. (2018) | The study achieved low risk of bias in three core technical domains. The only unclear risk is limited to the statistical analysis domain, arising from incomplete justification of normalization strategies and ambiguous reporting of multiple comparison adjustment for multi-metric analyses. |
| Pre-eclampsia | Zoe Brookes et al. (2025) | The study achieved low risk of bias in three core technical domains. The only unclear risk is limited to the statistical analysis domain, arising from incomplete justification of normalization strategies and ambiguous reporting of multiple comparison adjustment for multi-endpoint functional analyses. |
| Pre-eclampsia | Marloes Dekker Nitert et al. (2022) | The study strictly adhered to standardized microbiome research protocols across all four core assessment domains, with complete and transparent reporting of sampling, DNA extraction, 16S rRNA amplification, sequencing, QC, and bioinformatic analysis. All four core assessment domains rated as low risk of bias. The only minor limitation was the lack of direct public deposition of raw data, which did not alter the low overall risk of bias for the microbiome-specific methodology. |
| Pre-eclampsia | Marleen M. Kock et al. (2022) | The study strictly followed standardized microbiome research protocols for microbial measurement, bioinformatic analysis, and statistical testing, with complete reporting of sampling, DNA extraction, 16S rRNA amplification, bioinformatic pipeline parameters, and public data deposition. The only methodological limitation was the unclear description of raw sequencing read QC and the absence of explicit batch effect control strategies, which represent a minor deficiency without compromising the overall reliability and rigor of the study design and results. |
| Preterm birth | Irene Yang et al. (2022) | The study adheres to gold standards for microbiome research methodology across all critical domains. The only minor limitation is the lack of explicit batch effect correction documentation, which does not undermine the overall reliability, reproducibility, or validity of the study’s methodological design and results. |
| Preterm birth | Ju Sun Heo et al. (2024) | The study adheres to high standards of microbiome research methodology across all critical domains. The only minor deficiencies (lack of public data deposition and explicit batch effect correction documentation) do not compromise the overall reliability, reproducibility, or validity of the study’s methodological design and results. |
| Preterm birth | Anja Kovanda et al. (2023) | The study adheres to high standards of microbiome research methodology across all critical domains. The only minor deficiencies (lack of explicit batch effect correction documentation and unreported extraction/PCR controls) do not compromise the overall reliability, reproducibility, or validity of the study’s methodological design and results. |
| Preterm birth | Jin Kyu Kim et al. (2023) | The study adheres to high standards of microbiome research methodology across all critical domains. The only minor deficiencies (lack of explicit batch effect correction and unreported extraction/PCR controls) do not compromise the overall reliability, reproducibility, or validity of the study’s methodological design and results. |
| LBW | Yafei Wu et al. (2021) | The study adheres to high standards of microbiome research methodology across all critical domains. The only minor deficiencies (lack of explicit batch effect correction, unreported extraction/PCR controls, and univariate taxonomic comparisons) do not compromise the overall reliability, reproducibility, or validity of the study’s methodological design and results. |
| LBW | Pei Liu et al. (2024) | The study adheres to high standards of microbiome research methodology across all critical domains. The only minor deficiencies (unreported contamination controls and explicit batch correction) do not compromise the overall reliability, reproducibility, or validity of the methodological design. |
| Preterm birth+pre-eclampsia | Purnima S. Kumar et al. (2024) | The study represents a gold standard in microbiome research methodology. Strict contamination control, balanced groups, and advanced statistical methods (machine learning, source tracking, network analysis) enhance reliability. The only minor limitation (unreported batch correction) does not compromise the methodological rigor. |
| Adverse pregnancy outcomes | Rani Faryal et al. (2021) | The study’s core microbial measurement steps are valid and rigorous, but incomplete reporting of technical bias control, bioinformatic pipeline details, and microbiome-specific statistical rigor results in an overall unclear methodological quality with dominant unclear risk of bias across three key domains. |
| Adverse pregnancy outcomes | Rebecca Koerner et al. (2022) | This study demonstrates predominantly low risk of bias across all four core domains. Minor gaps (no explicit batch randomization, unmentioned public raw data deposition) do not compromise overall rigor, as technical bias is mitigated by longitudinal design and DADA2 processing, resulting in an overall high methodological quality with reliable, reproducible microbiome data. |
| Hypothyroidism | Yajuan Xu et al. (2020) | This study adheres to rigorous microbiome research standards, demonstrating low risk of bias in core methodological domains. Minor limitations (lack of explicit batch correction and multiple comparison adjustment) do not compromise the overall quality; the comprehensive, validated methodological framework provides credible evidence for oral/intestinal microbial differences between hypothyroid and normal pregnant women. |
| Stress and mental health symptoms | Joseph S Lonstein et al. (2024) | This study adheres to high standards of microbiome research methodology, demonstrating low risk of bias across core domains. Minor limitations (lack of explicit batch randomization, public raw data deposition) do not undermine the overall rigor. |
| Depressive symptoms | Sarven Sabunciyan et al. (2025) | This study adheres to high standards of microbiome research methodology, demonstrating low risk of bias across core domains. Minor limitations (lack of explicit batch randomization) do not undermine the overall rigor; the methodological framework ensures credible, reproducible findings on the association between the oral microbiome and antenatal depressive symptoms. |
| T2DM | Sophie M. Leech et al. (2025) | This study adheres to high standards of microbiome research methodology, demonstrating low risk of bias across core domains. Minor limitations (no explicit statistical adjustment for metformin use due to small sample size) is acknowledged by the authors and does not violate the low-risk criteria. |
| Arterial hypertension | Maria Joao ˜ Azevedo et al. (2025) | This study adheres to high standards of microbiome research methodology, demonstrating low risk of bias across core domains. Minor limitations (e.g., no explicit statistical batch correction, no public data deposition) are non-critical and do not violate the low-risk criteria. |

*Abbreviations: GDM: Gestational diabetes mellitus; LBW: Low birth weight; T2DM: Type 2 Diabetes Mellitus*
