# Supplementary material for: The oral microbiome in relation to systemic maternal conditions and pregnancy complications: a systematic review and meta-analysis
Source: J Oral Microbiol. 2026 Aug 26;18(1):2717774. doi: 10.1080/20002297.2026.2717774 (PMC13520856; doi:10.1080/20002297.2026.2717774)
Supplement: Supplementary file 6 [file ZJOM_A_2717774_SM2857.docx]

**Table S2. Association between oral microorganisms during pregnancy and adverse pregnancy outcomes.**

| **Author (year)** | **Study type** | **Disease** | **Microbial detection methods** | **Sample source** | **Sample collection time** | **Amplified region** | **Sequencing platform** | **Raw data availability** |
| --- | --- | --- | --- | --- | --- | --- | --- | --- |
| Xiu-Min Jiang et al. (2025) | Case control study | GDM | 16S rRNA sequencing | Saliva | Third trimester of pregnancy | (v3-v4) | Illumina NovaSeq 6000 | https://ngdc.cncb.ac.cn/gsa-human.  GSA-Human: HRA008585 |
| Jizhi Zhao et al. (2021) | Cross-sectional study | GDM | 16S rRNA sequencing | Subgingival plaque, saliva | 20-28 gestational weeks | (v4) | Illumina MiSeq | NR |
| Silvia Daher et al. (2018) | Cross-sectional study | GDM | 16S rRNA sequencing | Sterile swab | Third trimester of pregnant women | (v4) | Illumina MiSeq | SRA: SRP126329 |
| Mie K. W. Crusel et al. (2020) | Longitudinal study | GDM | 16S rRNA sequencing | Saliva | 27-33 gestational weeks | (v1-v3) | Illumina MiSeq | Not publicly available due to Danish Data Protection Agency |
| Yajuan Xu et al. (2019) | Case control study | GDM | 16S rRNA Sequencing | Saliva | Third trimester of pregnant women | (v3-v4) | Illumina Hiseq 2500 | https://doi.org/10.6084/m9.figshare.8977019.v1 |
| Jinfeng Wang et al. (2020) | Case control study | GDM | 16S rRNA Sequencing | Saliva and dental plaque | Third trimester of pregnant women | (v3-v4) | Illumina HiSeq2500 | GSA: CRA002189 |
| Fangqing Zhao et al. (2018) | Case control study | GDM | 16S rRNA sequencing | Saliva | 1-2 days before delivery | (v3-v4) | Illumina MiSeq | NR |
| Zoe Brookes et al. (2025) | Case control study | Pre-eclampsia | 16S rRNA sequencing | Saliva | Third trimester of pregnant women | (v3-v4) | Illumina MiSeq | NR |
| Marloes Dekker Nitert et al. (2022) | Case control study | Pre-eclampsia | 16S rRNA sequencing | Buccal swab | 36 weeks gestation | (v6-v8) | Illumina MiSeq | NR |
| Marleen M. Kock et al. (2022) | Cross-sectional study | Pre-eclampsia | 16S rRNA Sequencing | Saliva | 32-40 gestational weeks | (v3-v4) | Illumina MiSeq | SRA: PRJNA798597 |
| Rani Faryal et al. (2021)* | Cross-sectional study | Preterm birth, LBW, pre-eclampsia | 16S rRNA sequencing | Saliva | After first day of delivery | (v4) | Illumina MiSeq | SRA: PRJNA505914 |
| Rebecca Koerner et al. (2022)* | Case control study | Preterm birth, GDM, pre-eclampsia | 16S rRNA sequencing | Saliva | 17-19 gestational weeks, 25-27 gestational weeks, and 32-34 gestational weeks | (v4) | Illumina MiSeq | NR |
| Purnima S. Kumar et al. (2024)* | Case control study | Preterm birth, pre-eclampsia | whole-genome shotgun sequencing | Subgingival plaque and saliva | At the time of delivery | NA | Illumina HiSeq4000 | SRA of the NCBI database: PRJNA934596. |

*Abbreviations: GDM:* *Gestational diabetes mellitus; GSA: Genome Sequence Archive; LBW: Low birth weight; NCBI:* *National Center for Biotechnology Information; NR: not reported.* *SRA: Sequence Read Archives;* *rRNA: ribosomal RNA*

**Footnote:**

*** Rani Faryal et al. (2021) is listed in both Table S1 and Table S2 as it examined both preterm birth, low birth weight (birth outcome) and pre-eclampsia (pregnancy outcomes).**

***Rebecca Koerner et al. (2023) is listed in both Table S1 and Table S2 as it examined both preterm birth (birth outcome), pre-eclampsia and GDM (pregnancy outcomes).**

***Purnima S. Kumar et al. (2024) is listed in both TableS1 and Table S2 as the study examined both preterm birth (birth outcome) and pre-eclampsia (pregnancy outcome).**
