# Supplementary material for: The oral microbiome in relation to systemic maternal conditions and pregnancy complications: a systematic review and meta-analysis
Source: J Oral Microbiol. 2026 Aug 26;18(1):2717774. doi: 10.1080/20002297.2026.2717774 (PMC13520856; doi:10.1080/20002297.2026.2717774)
Supplement: Supplementary file 7 [file ZJOM_A_2717774_SM2858.docx]

**Table S3. Association between oral microorganisms during pregnancy and systemic diseases.**

| **Author(year)** | **Study type** | **Disease** | **Microbial detection methods** | **Sample source** | **Sample collection time** | **Amplified region** | **Sequencing platform** | **Raw data availability** |
| --- | --- | --- | --- | --- | --- | --- | --- | --- |
| Yajuan Xu et al. (2020) | Case control study | Hypothyroidism | 16S rRNA Sequencing | Saliva | Third trimester of pregnancy | (v3-v4) | Illumina Hiseq2500 | ENA: PRJEB38373 |
| Joseph S Lonstein et al. (2024) | Cross-sectional study | Stress and mental health symptoms | 16S rRNA Sequencing | Saliva | Second trimester (~17 ± 2 weeks gestation) | (v3-v4) | MiSeq system | NR |
| Sarven Sabunciyan et al. (2025) | Cross-sectional study | Depressive symptoms during pregnancy | 16S rRNA sequencing | Saliva | First-third trimester of pregnancy | (v3-v4) | NA | SRA: PRJNA1005610 |
| Sophie M. Leech et al. (2025) | Case control study | T2DM | Shotgun metagenomic sequencing | Buccal swab and Saliva rinse | 26-28 weeks of gestation; 34-38 weeks of gestation; | NA | Illumina NovaSeq 6000 | https://www.ncbi.nlm.nih.gov/sra/PRJNA1185841. PRJNA1185841 |
| Maria Joao ˜ Azevedo et al. (2025) | Prospective study | Arterial hypertension | 16S rRNA sequencing | Saliva | 3rd trimester of pregnancy, 24-28 weeks after delivery | (v4) | Illumina MiSeq | NR |

*Abbreviations: ENA: European Nucleotide Archive; NCBI: National Center for Biotechnology Information; NR: not reported; rRNA: ribosomal RNA; SRA: Sequence Read Archive; T2DM: Type 2 Diabetes Mellitus*
