# Supplementary material for: The oral microbiome in relation to systemic maternal conditions and pregnancy complications: a systematic review and meta-analysis
Source: J Oral Microbiol. 2026 Aug 26;18(1):2717774. doi: 10.1080/20002297.2026.2717774 (PMC13520856; doi:10.1080/20002297.2026.2717774)
Supplement: Supplementary file 8 [file ZJOM_A_2717774_SM2860.docx]

| **Table S7. Methodology and findings of the included studies assessing beta diversity.** | | | | |  |  |  |  |
| --- | --- | --- | --- | --- | --- | --- | --- | --- |
| **Study** | **Sample** | **Comparisons** | **Disease** | **Calculation** | **Visualization** | **Statistical method and threshold** | ***P* value** | **Finding** |
| Jinfeng Wang et al. (2020) | Saliva | GDM^+^ pregnant women VS GDM^-^ pregnant women | GDM | BrayCurtis, Unweighted UniFrac distance | PCoA | Mann-Whitney test (*P* < 0.05) | Not written | Sig. difference |
| Jinfeng Wang et al. (2020) | Dental plaque | GDM^+^ pregnant women VS GDM^-^ pregnant women | GDM | BrayCurtis, Unweighted UniFrac distance | PCoA | Mann-Whitney test (*P* < 0.05) | Not written | Sig. difference |
| Xiu-Min Jiang et al. (2025) | Saliva | GDM^+^ periodontis^+^ pregnant women VS GDM^+^ periodontis^-^pregnant women | GDM | Weighted UniFrac distances | PCoA | Mann-Whitney test (*P* < 0.05) | Not written | Sig. difference |
| Mie K. W. Crusel et al. (2020) | Saliva | GDM^+^ pregnant women VS GDM^-^ pregnant women | GDM | Weighted Unifrac distances | PCoA | PERMANOVA  (*P* < 0.05) | 0.58 | No. sig. difference |
| Silvia Daher et al. (2018) | Oral secretion sample by sterile swab | GDM^+^ pregnant women VS GDM^-^ pregnant women | GDM | Weighted and Unweighted Unifrac distances | PCoA | PERMANOVA  (*P* < 0.05) | *P* = 0.99 for Weighted UniFrac Analysis | No. sig. difference |
| Jizhi Zhao et al. (2021) | Dental plaque, saliva | GDM^+^ pregnant women VS GDM^-^ pregnant women | GDM | Weighted UniFrac distance | PCoA | AMOVA (*P* < 0.05) | Not written | No. sig. difference |
| Yajuan Xu et al. (2019) | Saliva | GDM^+^ pregnant women VS GDM^-^ pregnant women | GDM | BrayCurtis, Unweighted and Weighted UniFrac distance | NMDS | PERMANOVA (*P* < 0.05) | 0.3 | No. sig. difference |
| Rebecca Koerner et al. (2022) | Saliva | GDM^+^ pregnant women VS GDM^-^ pregnant women | GDM | Aitchison distances | NA | PERMANOVA(*P* < 0.05) | Not written | No. sig. difference |
| Marleen M. Kock et al. (2022) | Saliva | Pre-eclampsia^+^ pregnant women VS Pre-eclampsia ^-^ pregnant women | Pre-eclampsia | Weighted and Unweighted unifrac distance | PCoA | PERMANOVA(*P* < 0.05) | *P* for UniFrac weighted = 0.354; *P* for Unweighted = 0.185 | No. sig. difference |
| Marloes Dekker Nitert et al. (2022) | Oral swab from buccal surface | Pre-eclampsia^+^ pregnant women VS Pre-eclampsia ^-^ pregnant women | Pre-eclampsia | BrayCurtis distance | PCoA, RDA | ANOSIM (*P* < 0.05) | 0.045 | Sig. difference |
| Zoe Brookes et al. (2025) | Saliva | Pre-eclampsia^+^ pregnant women VS Pre-eclampsia ^-^ pregnant women | Pre-eclampsia | Aitchison distances | Multidimensional scaling ordination | Mixed-effect linear models (*P* < 0.05) | Not written | No. sig. difference |
| Rebecca Koerner et al. (2022) | Saliva | Pre-eclampsia^+^ pregnant women VS Pre-eclampsia ^-^ pregnant women | Pre-eclampsia | Aitchison distances | NA | PERMANOVA  (*P* < 0.05) | 0.015 | Sig. difference |
| Yafei Wu et al. (2021) | Subgingival plaque | PLBW^+^periodontis^+^ pregnant women VS PLBW^+^periodontis^-^ pregnant women VS PLBW^-^periodontis^+^ pregnant women VS PLBW^-^periodontis^-^ pregnant women | PLBW | BrayCurtis, Unweighted UniFrac distance metrics | PCA | Not written | 0.121 | No. sig. difference |
| Irene Yang et al. (2022) | Subgingival plaque | Spontaneously delivered early term VS Full term birth | Preterm birth | BrayCurtis, Jaccard | PCoA | PERMANOVA  (*P* < 0.05) | Not written | Sig. difference |
| Jin Kyu Kim et al. (2023) | Saliva | Preterm birth VS Full term birth | Preterm birth | Hamming distance | NA | PERMANOVA  (*P* < 0.05) | 0.908 | No. sig. difference |
| Ju Sun Heo et al. (2024) | Subgingival plaque | Preterm birth VS Full term birth | Preterm birth | BrayCurtis, Weighted Unifrac distance, Jaccard | PCoA | PERMANOVA (*P* < 0.05) | *P* for Weighted unifrac = 0.3128; *P* for Bray-curtis = 0.2346; *P* for Jaccard = 0.3279 | No. sig. difference |
| Anja Kovanda et al. (2023) | A pooled saliva, tongue, hard palate, and buccal mucosa swab. | Preterm birth VS Full term birth | Preterm birth | Weighted Unifrac distance | PCoA | Not written | Not written | No. sig. difference |
| Rebecca Koerner et al. (2022) | Saliva | Preterm birth pregnant women VS Full term birth pregnant women | Preterm birth | Aitchison distances | NA | PERMANOVA  (*P* < 0.05) | Not written | No. sig. difference |
| Rani Faryal et al. (2021) | Saliva | Full term infant delivery females VS PLBW infant delivery females | PLBW | BrayCurtis | NA | Not written | BC = 0.55 | Sig. difference |
| Pei Liu et al. (2024) | Saliva | Low birth weight infant delivery females VS Normal birth weight infant delivery females | Low weight birth | Unweighted UniFrac | PCoA | Not written | 0.305 | No. sig. difference |
| Yajuan Xu et al. (2020) | Saliva | Pregnant women with hypothyroidism VS Normal pregnant women | Hypothyroidism | Weighted Unifrac distances | PCoA | ANOSIM | *P*＜0.05 | Sig. difference |
| Maria Joao ˜ Azevedo et al. (2025) | Saliva | Pregnant women with arterial hypertension VS Healthy pregnant women | Arterial hypertension | Aitchison distances | PCA | PERMANOVA | *P* for third trimester = 0.02; *P* for six months after delivery = 0.03 | Sig. difference |
| Sophie M. Leech et al. (2025) | Saliva rinse | Pregnant women with preexisting T2DM VS Pregnant normoglycaemic controls | T2DM | Aitchison distances | Not written | *Adonis2* | Not written | No. sig. difference |
| Sophie M. Leech et al. (2025) | Swab | Pregnant women with preexisting T2DM VS Pregnant normoglycaemic controls | T2DM | Aitchison distances | Not written | *Adonis2*  (*P* < 0.05) | Not written | No. sig. difference |
| Joseph S Lonstein et al. (2024) | Saliva | High PTSD symptoms VS Low PTSD symptoms | PTSD | BrayCurtis, Weighted unifrac | PCoA | PERMANOVA  (*P* < 0.05) | *P* for Weighted UniFrac = 0.037 | Sig. difference |
| Joseph S Lonstein et al. (2024) | Saliva | High stress symptoms VS Low stress symptoms | Stress | BrayCurtis, Weighted unifrac | PCoA | PERMANOVA  (*P* < 0.05) | *P* for Weighted UniFrac = 0.051 | No. sig. difference |
| Joseph S Lonstein et al. (2024) | Saliva | High anxiety symptoms VS Low anxiety symptoms | Anxiety | BrayCurtis, Weighted unifrac | PCoA | PERMANOVA  (*P* < 0.05) | *P* for Weighted UniFrac = 0.429 | No. sig. difference |
| Joseph S Lonstein et al. (2024) | Saliva | High depression symptoms VS Low depression symptoms | Depression | BrayCurtis, Weighted unifrac | PCoA | PERMANOVA  (*P* < 0.05) | *P* for Weighted UniFrac = 0.321 | No. sig. difference |
| Sarven Sabunciyan et al. (2025) | Saliva | Women with depressive symptoms during pregnancy VS Women without depressive symptoms during pregnancy | Depression | BrayCurtis, Euclidean distance: | PCA | PERMANOVA  (*P* < 0.05) | 0.575 | No. sig. difference |

*Abbreviations: AMOVA, Analysis of Molecular Variance; ANOSIM, Analysis of similarities; GDM, Gestational diabetes mellitus; NMDS, Non-metric multidimensional scaling; PERMANOVA, Permutational analysis of variance; PCA, Principal component analysis; PCoA, Principal coordinates analysis; PLBW, Preterm low birth weight; PTSD, Post-traumatic stress disorder; RDA, Redundancy analysis; T2DM, Type 2 Diabetes Mellitus.*
