# Supplementary material for: The oral microbiome in relation to systemic maternal conditions and pregnancy complications: a systematic review and meta-analysis
Source: J Oral Microbiol. 2026 Aug 26;18(1):2717774. doi: 10.1080/20002297.2026.2717774 (PMC13520856; doi:10.1080/20002297.2026.2717774)
Supplement: Supplementary file 9 [file ZJOM_A_2717774_SM2861.docx]

| **Table S6. Risk of bias assessment for microbiome-specific methodologies in the study.** | | | | | | |
| --- | --- | --- | --- | --- | --- | --- |
| Study ID | Disease | Microbial measurement reliability | Sequencing and batch effect control | Bioinformatic pipeline consistency | Statistical analysis appropriateness | Overall quality |
| Jinfeng Wang et al. (2020) | GDM | Low | Low | Low | Low | Low |
| Xiu-Min Jiang et al. (2025) | GDM | Low | Low | Low | Unclear | Low |
| Jizhi Zhao et al. (2021) | GDM | Unclear | Unclear | Low | Unclear | Unclear |
| Silvia Daher et al. (2018) | GDM | Low | Low | Low | Unclear | Low |
| Mie K. W. Crusel et al. (2020) | GDM | Low | Low | Low | Unclear | Low |
| Yajuan Xu et al. (2019) | GDM | Low | Unclear | Low | Unclear | Unclear |
| Fangqing Zhao et al. (2018) | GDM | Low | Low | Low | Unclear | Low |
| Zoe Brookes et al. (2025) | Pre-eclampsia | Low | Low | Low | Unclear | Low |
| Marloes Dekker Nitert et al. (2022) | Pre-eclampsia | Low | Low | Low | Low | Low |
| Marleen M. Kock et al. (2022) | Pre-eclampsia | Low | Unclear | Low | Low | Low |
| Irene Yang et al. (2022) | Preterm birth | Low | Low | Low | Low | Low |
| Ju Sun Heo et al. (2024) | Preterm birth | Low | Low | Low | Low | Low |
| Anja Kovanda et al. (2023) | Preterm birth | Low | Low | Low | Low | Low |
| Jin Kyu Kim et al. (2023) | Preterm birth | Low | Low | Low | Low | Low |
| Yafei Wu et al. (2021) | LBW | Low | Low | Low | Low | Low |
| Pei Liu et al. (2024) | LBW | Low | Low | Low | Low | Low |
| Purnima S. Kumar et al. (2024) | Preterm birth, pre-eclampsia | Low | Low | Low | Low | Low |
| Rani Faryal et al. (2021) | Preterm birth, LBW, pre-eclampsia | Low | Unclear | Unclear | Unclear | Unclear |
| Rebecca Koerner et al. (2022) | Preterm birth, GDM pre-eclampsia | Low | Low | Low | Low | Low |
| Yajuan Xu et al. (2020) | Hypothyroidism | Low | Low | Low | Low | Low |
| Joseph S Lonstein et al. (2024) | Stress and mental health symptoms | Low | Low | Low | Low | Low |
| Sarven Sabunciyan et al. (2025) | Depressive symptoms | Low | Low | Low | Low | Low |
| Sophie M. Leech et al. (2025) | T2DM | Low | Low | Low | Low | Low |
| Maria Joao ˜ Azevedo et al. (2025) | Arterial hypertension | Low | Low | Low | Low | Low |

*Abbreviations: GDM: Gestational diabetes mellitus; LBW: Low birth weight; T2DM: Type 2 Diabetes Mellitus*
