# Supplementary material for: The oral microbiome in relation to systemic maternal conditions and pregnancy complications: a systematic review and meta-analysis
Source: J Oral Microbiol. 2026 Aug 26;18(1):2717774. doi: 10.1080/20002297.2026.2717774 (PMC13520856; doi:10.1080/20002297.2026.2717774)
Supplement: Supplementary file 12 [file ZJOM_A_2717774_SM2864.docx]

**Appendix S1. Electronic search strategy in PubMed, Embase and Web of Science.**

**PubMed Strategy**

("Mouth"[Mesh] OR "Saliva"[Mesh] OR "Gingival Crevicular Fluid"[Mesh] OR "Mouth"[tiab] OR "Mouths"[tiab] OR "Dental"[tiab] OR "Teeth"[tiab] OR "Tooth"[tiab] OR "Periodont*"[tiab] OR "Gingiv*"[tiab] OR "Subgingiv*"[tiab] OR "Supragingiv*"[tiab] OR "Gum"[tiab] OR "Gums"[tiab] OR "Caries"[tiab] OR "Saliv*"[tiab]) AND ("Microbiota"[Mesh] OR "Microbiot*"[tiab] OR "Micro-biot*"[tiab] OR "Microbiom*"[tiab] OR "Metagenomic*"[tiab] OR "Microflor*"[tiab] OR "Flora"[tiab]) OR "Bacter*"[tiab] OR "Microb*"[tiab]) AND ("High-Throughput Nucleotide Sequencing"[Mesh] OR 16S[tiab] OR Sequencing[tiab] OR Shotgun[tiab] OR Metagenome[tiab] OR NGS[tiab] OR illumina [tiab]) AND ("Pregnancy"[Mesh] OR "Pregnant Women"[Mesh] OR "Pregnancy Trimesters"[Mesh] OR "Pregnancy Outcome"[Mesh] OR "Pregnancy Complications"[Mesh] OR "Obstetric Labor Complications"[Mesh] OR "Premature Birth"[Mesh] OR "Pre-Eclampsia"[Mesh] OR "Stillbirth*"[tiab] OR "Still birth*"[tiab] OR "Spontaneous Abortion*"[tiab] OR "Pregnancy Outcome*"[tiab] OR "Birth Outcome*"[tiab] OR "Labor Outcome*"[tiab] OR "Labour Outcome*"[tiab] OR "Obstetric*"[tiab] OR "Pre Eclamp*"[tiab] OR "Preeclamp*"[tiab] OR "Preclamp*"[tiab])NOT ("Animals"[Mesh] NOT ("Animals"[Mesh] AND "Humans"[Mesh])) NOT ("Review" [Publication Type] OR "Letter" [Publication Type] OR "Editorial" [Publication Type] OR "Case Reports" [Publication Type])

**Embase Strategy**

('mouth'/exp OR 'saliva'/exp OR 'mouth disease'/exp OR 'periodontal index'/exp OR 'dmft index'/exp OR (mouth* OR dental OR teeth OR tooth OR periodont* OR gingiv* OR subgingiv* OR supragingiv* OR gum OR gums OR caries OR saliv*):ti,ab) AND ('microflora'/exp OR microbiot* OR micro-biot* OR biota OR microbiom*OR microflor* OR flora) AND ('High-Throughput Nucleotide Sequencing':ti,ab,kw OR '16S':ti,ab,kw OR 'Sequencing':ti,ab,kw OR 'Shotgun':ti,ab,kw OR 'Metagenome':ti,ab,kw OR 'NGS':ti,ab,kw OR 'illumina':ti,ab,kw OR 'pyrosequencing':ti,ab,kw) AND ('pregnancy'/exp OR 'pregnant woman'/exp OR 'pregnancy outcome'/exp OR 'pregnancy complication'/exp OR 'labor complication'/exp OR 'prematurity'/exp OR 'preeclampsia'/exp OR early pregnancy-loss* OR miscarriage* OR pregnancy-outcome* OR birth-outcome* OR labor-outcome* OR labour-outcome* OR pre-eclamp* OR preeclamp* OR preclamp*) NOT ([animals]/lim NOT [humans]/lim) AND ('article'/it OR 'article in press'/it OR 'chapter'/it OR 'conference paper'/it OR 'erratum'/it OR 'short survey'/it)

**Web of Science Strategy**

TS=(mouth OR saliva OR mouth-disease OR periodont* OR gingiv* OR subgingiv* OR supragingiv*) AND TS=(microflora OR microbiomes OR microbiot* OR microbiome OR biota OR microbiom* OR microflor*) AND TS=(High-Throughput Nucleotide Sequencing OR 16S OR Sequencing OR Shotgun OR Metagenome OR NGS OR illumina OR pyrosequencing) AND TS=(pregnancy OR pregnant woman OR pregnancy outcome OR pregnancy complication OR labor complication OR prematurity OR preeclampsia OR pregnan* OR prenatal-care OR stillbirth* OR still-birth* OR spontaneous-abortion* OR early-pregnancy-loss* OR miscarriage* OR pregnancy-outcome* OR birth-outcome* OR labor outcome* OR labour-outcome* OR obstetric* OR pre-eclamp* OR preeclamp* OR preclamp*) Refined by: [excluding] DOCUMENT TYPES: ( PATENT OR EDITORIAL MATERIAL OR REVIEW OR CASE REPORT OR ABSTRACT OR LETTER OR MEETING )
