# Supplementary material for: The oral microbiome in relation to systemic maternal conditions and pregnancy complications: a systematic review and meta-analysis
Source: J Oral Microbiol. 2026 Aug 26;18(1):2717774. doi: 10.1080/20002297.2026.2717774 (PMC13520856; doi:10.1080/20002297.2026.2717774)
Supplement: Supplementary file 13 [file ZJOM_A_2717774_SM7829.docx]

**Supplementary materials**

Figure S1. Forest plots of alpha diversity estimators in the oral microbiota of pregnant women with gestational diabetes mellitus (GDM) compared with controls. (A) Observed richness (B) ACE index (C) Chao1 index (D) Simpson index (E) Shannon index

Figure S2. Forest plots of alpha diversity estimators in the oral microbiota of pregnant women with low birth weight (LBW) compared with controls. (A) Chao1 index (B) Shannon index

Figure S3. Forest plots of alpha diversity estimators in the oral microbiota of pregnant women with pre-eclampsia compared with controls. (A) Shannon index

Figure S4. Forest plots of alpha diversity estimators in the oral microbiota of pregnant women with preterm birth compared with controls.(A) Simpson index (B) Chao1 index
